# Coordinated self-interference of wave packets: a new route towards classicality for structurally stable systems

**DOI:** 10.1038/s41598-020-72965-w

**Published:** 2020-10-12

**Authors:** M. Ćosić, S. Petrović, S. Bellucci

**Affiliations:** 1 Laboratory of Physics, Vinča Institute of Nuclear Sciences, University of Belgrade, P. O. Box 522, 11001 Belgrade, Serbia; 2 INFN-Laboratori Nazionali di Frascati, Frascati, 1-00044 Rome, Italy

**Keywords:** Atomic and molecular collision processes, Quantum mechanics, Theoretical physics, Nonlinear phenomena

## Abstract

This is a study of proton transmission through planar channels of tungsten, where a proton beam is treated as an ensemble of noninteracting wave packets. For this system, the structural stability manifests in an appearance of caustic lines, and as an equivalence of self-interference produced waveforms with canonical diffraction patterns. We will show that coordination between particle self-interference is an additional manifestation of the structural stability existing only in ensembles. The main focus of the analysis was on the ability of the coordination to produce classical structures. We have found that the structures produced by the self-interference are organized in a very different manner. The coordination can enhance or suppress the quantum aspects of the dynamics. This behavior is explained by distributions of inflection, undulation, and singular points of the ensemble phase function, and their bifurcations. We have shown that the coordination has a topological origin which allows classical and quantum levels of reality to exist simultaneously. The classical behavior of the ensemble emerges out of the quantum dynamics without a need for reduction of the quantum to the classical laws of motion.

## Introduction

A state of a classical particle is completely determined by specifying its current position and linear momentum. The state space of the system is a multidimensional manifold called phase space. An evolution of the classical system is specified by mappings transforming the phase space onto itself^1^.

The state space of a quantum system is a more abstract Hilbert space of square-integrable functions, bearing no direct relationship to the phase space. An evolution of the quantum system is specified by a combination of non-commuting operators mapping Hilbert space onto itself^2,3^. An additional requirement of the quantum theory is that quantum and classical mechanics must be connected in a way that allows information about the quantum system to be encoded in the classical dynamics of a measuring apparatus making it accessible to an observer. This concept, known as the correspondence principle, is one of the cornerstones of the *Copenhagen* interpretation of quantum mechanics^4^.

During the development of quantum mechanics, the correspondence principle was stated in several ways. The earliest formulation, given by Bohr, claims that in the limit of large quantum numbers predictions of the quantum approach coincide with predictions of the classical approach. A modern formulation of this principle is slightly different. Namely, classical structures emerge when the number of excited quantum states is large. An example of such behavior occurs in high energy scattering when a number of partial waves, contributing significantly to the scattering process, is large^5^. Another example is a reduction of a high energy many-beam diffraction to a classical bounded motion of particles in potential wells of atomic strings or planes, know as channeling^6^. A related concept is, so-called, the short wavelength limit (or a semiclassical limit) where it is claimed that classical features emerge out of the quantum dynamics when de Broglie’s wavelength $\lambda _{dB}$ of particle tends to zero^7^.

Although seemingly simple on the surface it is highly subtle. The semiclassical analysis applies in the limit of vanishing ratio of reduced Planck’s constant $\hbar$ and corresponding classical action, which is mathematically equivalent to the limit $\hbar \rightarrow 0$. However, limiting value $\hbar =0$ is singular since it annuls terms of the Schrödinger equation responsible for spatio–temporal variations of the wave function, thus making it physically meaningless. An additional difficulty arises when a classical dynamics allows several particles to arrive at the same place, and produce a multivalued velocity field. In that case, a domain containing classical trajectories can be partitioned according to the multiplicity of its points. Lines separating different subdomains, known as caustics, are of great importance since along them a density of particle trajectories is infinite. They are responsible of all manifestations of optical^8^, atomic^9^, nuclear^10^, surface^11^, and a crystal rainbow effects^12^. On the other hand, the quantum velocity field must be single-valued. Therefore, investigation of the semiclassical limit requires great care in handling multiplicities of the classical velocity field and its associated singularities^7,13^.

All mentioned technical difficulties can be surpassed by the use of asymptotic analysis^14^ aided by uniform approximation techniques^15^, or group contractions^16^. However, the main conceptual difficulty, associated with the unremovable singularity $\hbar =0$, remains. The classical limit is not achievable without some averaging process imposed externally^17,18^. This drawback has been known from the times of Thomas Young and it is related to his famous double-slit experiment. Namely, in the short wavelength limit oscillations of the light intensity, caused by the interference, become infinitely fast. The theory is incapable to explain why a combined light intensity of two identical classical light sources is not a simple sum of individual intensities. To get around this difficulty it is necessary to assume that any practical detector of light has finite bandwidth. As a consequence, its measuring output is sensitive only to the mean value of measuring signal. This means that classical reality is created by a process of observation—a concept known as the wave function collapse in the *Copenhagen* interpretation of quantum mechanics.

Needless to say that a lot of scientists find this implication troublesome. To understand better the connection between quantum and classical physics scientists tried to reformulate classical mechanic in the terms of commuting operators acting on appropriate Hilbert space like in Koopman–von Neumann’s approach^19,20^ or tried to find the phase space formulation of the quantum mechanics in which the use of the operator formalism can be avoided^21–23^.

Some researchers embraced the idea that interaction is necessary for the production of classical reality. A major insight of a decoherence theory^24^ stems from the observation that there are no quantum systems that are fully isolated from an environment. When conditions are such that interaction with the environment is strong it behaves as a continuous monitoring apparatus forcing the quantum system to be all the time in the well-defined state. A merit of such an approach is that the much objectionable wave function collapse is a physical process independent of the observer. It also explains why observation of large-scale quantum interference effects requires the use of sophisticated scientific equipment.

It should be said that all described considerations are quite general, applicable to all quantum systems. Here we will focus on properties of structurally stable systems that have a special property that their topological (or morphological) features are unaffected by a relatively large variation of system parameters. When the morphology of the system does change, it happens abruptly for very small changes of critical values of parameters^25–27^. Therefore, the topology constraints a number of ways in which the dynamic of a structurally stable system can unfold. This deliberate reduction in scope is not a serious drawback since the vast majority of physical systems are structurally stable. It will be shown that there exists an additional route towards classicality, available to this kind of systems.

Here we will investigate the ensemble of noninteracting quantum packets and try to find the conditions under which classical and the quantum levels of reality exist simultaneously. Note that in the standard approach the quantum-classical correspondence is studied through the limit $\hbar \rightarrow 0$. In those studies, scientists are usually investigating a manner in which the behavior of a single quantum particle becomes classical. Another approach worth mentioning is that the classical behavior of the quantum ensemble emerges out of the dynamics of coherent states, which are rather special. They are represented by non-spreading wave packets whose probability densities are concentrated around corresponding classical trajectories^28^. In essence, they are classical as much as they are quantum. However, it is not an easy task to find coherent states for arbitrary potential and it is an open question whether it is always possible to prepare such states in a laboratory (see discussion in Refs.^29–31^).

In all mentioned studies it is meaningless to explore the classical limit of the ensemble since its classicality is a trivial consequence of the classicality of ensemble members. We will investigate an additional possibility, not explored by other researchers, and show that classical structures can emerge on the level of the ensemble out of purely quantum dynamics of ensemble members. This *morphological* quantum-classical transition is not related to any particular value of a ratio of a classical action and reduced Planck’s constant.

The *morphological* transition should appear in particle interference experiments, where only one particle is traversing any arm of the interferometer at any instance of time^32^, or in a motion of atoms or ions in potentials of optical lattices or ion traps^33^. Another example easily accessible for experimental investigation is particle channeling through thin nanostructured materials in the regime of low current where each particle is transmitting through the sample independently^34–36^.

We will investigate the transmission of protons, represented as wave packets, through the [111] planar channels of W crystal. The $yoz$ plane of the Cartesian coordinate system is parallel to the [111] planes of the tungsten crystal. Quasi-parallel proton beam of kinetic energy $E_k=2$MeV was assumed to be aligned with the $z$-axis of the coordinate system, and let $k_z$ be a longitudinal component of the wave vector. Using Molière’s potential the continuous potential of the Tungsten [111] plane can be written in the form

1 $$\begin{aligned} \begin{array}{lcl}V_{111}^{\text {th}}(x)={\bar{V}}_0\sum\limits_{n=1}^3 \frac{\alpha _n}{\beta _n}\exp \left[ \frac{\beta _n^2\sigma _{\rm {th}}^2}{2a_{\rm {s}}^2}\right] {}\times \left\{ \exp \left[ -\frac{\beta _n|x|}{a_{\rm {s}}}\right] \text {erfc}\left( \frac{\beta _n\sigma _{{\rm {th}}}}{\sqrt{2}a_{\rm {s}}}- \frac{|x|}{\sqrt{2}\sigma _{\rm {th}}}\right) +\exp \left[ \frac{\beta _n|x|}{a_{\rm {s}}}\right] \text {erfc}\left( \frac{\beta _n\sigma _{\rm {th}}}{\sqrt{2}a_{\rm {s}}}+ \frac{|x|}{\sqrt{2}\sigma _{\rm {th}}}\right) \right\} , \end{array} \end{aligned}$$

where ${\bar{V}}_0$ is amplitude of the potential, $a_{\text {s}}$ is the screening length, $\sigma _{\rm {th}}$ is amplitude of the tungsten’s atoms thermal vibrations, $\varvec{\alpha }=(0.35, 0.55, 0.1)$ and $\varvec{\beta }=(0.3, 1.2, 6)$ are dimensionless Molière’s fitting parameters, while $\text {erfc}$ is complementary error function^37^. Potential of the planar channel is a sum of contributions of atomic planes located at distances $d_m=(m+\frac{1}{2})d_{111}$ from the coordinate origin, here $d_{111}$ stands for the distance between neighboring [111] panes. The resulting potential of the channel is given by the following expression

2 $$\begin{aligned} V(x) = \sum _{m=0}^\infty \left( V^{\rm {th}}_{111}(x+d_m)+V^{\rm {th}}_{111}(x-d_m)\right) -V_0. \end{aligned}$$

Constant $V_0=2\sum _m V^{\rm {th}}_{111}(d_m)$ was introduced in order to have $V(0)=0$. The maximal scattering angle channeled particle could have, called the critical channeling angle, is defined by the following expression $\Theta _c=(V(d_{111}/2)/E_k)^{1/2}$.

Dynamics in the longitudinal direction is trivial and will be neglected from further analysis. In the transverse direction proton dynamics of the classical particle is governed by Hamilton’s equations of motion

3 $$\begin{aligned} \frac{\text {d}}{\text {d}t} \theta _x= -\frac{\partial _x V(x)}{\sqrt{2m_\text {p}E_k}},\quad \frac{\text {d}}{\text {d}t}x = \sqrt{2\frac{E_k}{m_\text {p}}}\theta _x, \end{aligned}$$

where $\theta _x$ is proton scattering angle, $m_\text {p}$ is proton mass while $t$ denotes time. The dynamics of quantum state $\psi (x,t)$ is governed by the Schrödinger equation

4 $$\begin{aligned} i\hbar \partial _t\psi (x,t) = \left[ -\frac{\hbar ^2}{2m_\text {p}}\partial ^2_x+V(x)\right] \psi (x,t). \end{aligned}$$

To represent parallel beam initial conditions for classical particles should be in the form $\theta _x(t=0)=0$, and $x(t=0)=b$, with uniform distribution of the proton impact parameters $b$. In the quantum approach initial state of the proton is given by a Gaussian wave packet

5 $$\begin{aligned} \psi _b(x)=\frac{1}{\sqrt{\sqrt{2\pi \sigma _x^2}}}\exp \left[ -\frac{(x-b)^2}{4\sigma _x^2}\right] , \end{aligned}$$

of mean value $b$, and standard deviation $\sigma _x$. The uniform distribution of impact parameters gives a quasi-parallel proton beam of angular divergence inversely proportional to the $\sigma _x$. For convenience, both classical and quantum dynamics will be parameterized by the variable $\Lambda =\frac{1}{2\pi }\omega t$ called reduced time,^33^ or reduced crystal thickness^38^. Here a variable

6 $$\begin{aligned} \omega =\left. \sqrt{\frac{\partial _x^2V(x)}{m_\text {p}}}\right| _{x=0}, \end{aligned}$$

represents the angular frequency of the proton trajectories near a center of the potential well. Details of the theoretical model can be found in the supplementary material.

Since the interaction between protons and crystal’s electrons can lead to decoherence, our analysis will be limited to the transmission through very thin crystals where proton–electron interaction is negligible. The main goal will be to indicate which physical mechanisms lead to amplification or suppression of the quantum nature of the ensemble. To do so, our analysis will be focused on the morphological properties of a Wigner function defined by the integral^21^

7 $$\begin{aligned} W_b(x,\theta _x)=\frac{k_z}{2\pi }\int \psi _b^\dag \left( x-\frac{\xi }{2}\right) \psi _b\left( x+\frac{\xi }{2}\right) \exp \left[ -ik_z\theta _x\xi \right] \text {d}\xi , \end{aligned}$$

and its catastrophes, i.e. places in the phase space where Wigner function locally oscillates with a large amplitude^39,40^. Wigner functions have been used extensively for investigations of the similarities and differences between classical and quantum dynamics. It is defined as a Weyl transformation of the density matrix operator, and it represents the closest quantum analog of the classical phase space probability density, although it can not be the true density since it can assume negative values. The negativity of the Wigner function is usually viewed as an undesirable property that should be eliminated by filtering as in the definition of Husimi *Q*-function^41^. Here we take the opposite view. We embrace it and use it to quantify how prominent interference effects are. Negativity is here used similarly as it is used to indicate the level of the decoherence^24^. In our opinion presented analysis is very important for understanding the subtleties of the classical-quantum correspondence. In essence, we are investigating the change of system dynamics resulting from the change of its parameters. Our geometrical analysis applies to all structurally stable systems. On the practical level, the presented results are relevant for all systems where second-order interference is important.

## Methods

For any fixed value of $\Lambda$ family of classical trajectories define maps of a particle’s starting position $b$ to its current position $X(b)\equiv x(\Lambda ;b)$, and current scattering angle $\Theta _x(b)\equiv \theta _x(\Lambda ;b)$, respectively. Considered together, maps $X$($b$), and $\Theta _x(b)$ define a curve in the phase space ${\mathcal {R}}=(Q(b),P(b))$, called a rainbow diagram^42^ or a whorl^43^, whose critical points are spatial and angular rainbows. Critical points $\partial _b x(\Lambda ; b)=0$ and $\partial _b \theta _x(\Lambda ;b)=0$ form lines in $(\Lambda ,x)$ and $(\Lambda ,\theta _x)$ spaces called caustics^44^. Geometrically they are envelope lines associated with respective trajectory families^45^. The density of trajectories is infinite on caustic lines. Therefore, the probability of finding a classical particle on the caustic line, or at the rainbow point is very large.

The wave function in the angular representation $\varphi (\theta _x,\Lambda )$ is defined by the integral

8 $$\begin{aligned} \varphi (\theta _x,\Lambda ) = \sqrt{\frac{k_z}{2\pi }}\int \psi (x,\Lambda )\exp \left[ -ik_z\theta _xx\right] \text {d}x. \end{aligned}$$

In the angular representation, the initial wave function is also given by a Gaussian function

9 $$\begin{aligned} \varphi (\theta _x,0)=\frac{1}{\sqrt{\sqrt{2\pi \sigma _\theta ^2}}}\exp \left[ -\frac{\theta _x^2}{4\sigma _\theta ^2}-ik_z\theta _xb\right] , \end{aligned}$$

where parameters $\sigma _x$ and $\sigma _\theta$ are linked through Heisenberg’s uncertainty relation $k_z\sigma _\theta \sigma _x=1/2$.

Hamilton’s principal function in the angular representation is defined by the equation

10 $$\begin{aligned} \frac{\text {d}}{\text {d}\theta _x} S_\theta (\theta _x) =-\hbar k_zx(\theta _x). \end{aligned}$$

Let us introduce a reduced principal function ${{\bar{S}}}_\theta$ by the relation $S_\theta (\theta _x)=-\hbar k_z{{\bar{S}}}_\theta (\theta _x)$. In the initial value representation^46,47^, a semiclassical wave function is given by the integral

11 $$\begin{aligned} \begin{array}{c} \psi (x,\Lambda ) = \frac{1}{\sqrt{2\pi }}\int |\psi (b,0)|\exp \left[ -ik_z\left( {\bar{S}}_\theta (b)+x\Theta _x(b)\right) \right] \sqrt{\frac{\text {d}\Theta _x(b)}{\text {d}b}}\text {d}b, \end{array} \end{aligned}$$

where $\text {d}{{\bar{S}}}_\theta (b) =X(b)\text {d}\Theta _x(b)$. Validity of the semiclassical representation (11) requires $k_zd_{111}\gg 1$. The proton beam is represented by an ensemble of noninteracting wave packets where states $\psi _b(x,\Lambda )$ of the ensemble are parameterized by the impact parameter $b$. The state of the ensemble is specified by the density matrix operator ${\hat{\rho }}$, whose spatial and angular representations, $\rho _x$, and $\rho _\theta$, are given by relations

12 $$\begin{aligned} \rho _x(x,\Lambda )=\sum _b p_b|\psi _b(x,\Lambda )|^2,\quad \rho _\theta (\theta _x,\Lambda )=\sum _b p_b|\varphi _b(\theta _x,\Lambda )|^2, \end{aligned}$$

respectively. Expansion coefficients $p_b$ represent the relative frequency of the state $\psi _b$ in the ensemble ($\sum _b p_b =1$). The incoming beam was assumed to be a Gaussian of very small angular standard deviation $\Omega$. Unknown parameters $\sigma _\theta$, $\sigma _x$, the distribution of the impact parameters, and statistical weights $p_b$, should be determined in such a way that $\rho _x(x,0)$ represents uniform distribution in the interval of length $d_{111}$, while $\rho _\theta (\theta _x,0)$ is

13 $$\begin{aligned} \rho _\theta (\theta _x,0) = \frac{1}{\sqrt{2\pi \Omega ^2}}\exp \left[ -\frac{\theta _x^2}{2\Omega ^2}\right] . \end{aligned}$$

Using Eqs. (9) and (12) it is easy to show that $\sigma _\theta =\Omega$, giving $\sigma _x = 1/(2k_z\Omega )$. An 1D grid of $M$ impact parameters was taken to uniformly cover the interval $-d_{111}/2\le x\le d_{111}/2$, with $p_b=1/M$. The number *M* is minimal for which the difference between $\rho _x(x,0)$ and a corresponding uniform distribution is smaller than some predetermined quantity. Wigner function of the ensemble is given by the expression

14 $$\begin{aligned} W(x,\theta _x) = \sum _b p_b W_b(x,\theta _x). \end{aligned}$$

The caustic pattern is structurally stable^7, 27, 48^. This means that it can be modeled locally by an appropriate catastrophic prototype of codimension one

15 $$\begin{aligned} A_k(\eta ;c_1,c_2,\ldots ,c_{k-1}) = \frac{1}{k+1}\eta ^{k+1}+\frac{c_{k-1}}{k-1}\eta ^{k-1}+\cdots +\frac{c_{2}}{2}\eta ^{2}+c_1\eta . \end{aligned}$$

In the quantum domain multiplicities of maps $X\rightarrow b$ and $\Theta _x\rightarrow b$, are responsible for the enhancement of the wave packet self-interference in the vicinity of caustic lines or rainbow diagram^7,33,49^. Resulting interference patterns are locally isomorphic to structurally stable canonical diffraction patters defined by the relation^7,27^

16 $$\begin{aligned} \chi _k(c_1,c_2,\ldots ,c_{k-1})=\frac{1}{\sqrt{2\pi }}\int \exp \left[ iA_k(\eta ;c_1,c_2,\ldots ,c_{k-1})\right] \text {d}\eta . \end{aligned}$$

In the classical approach, Hamilton’s equations (3) were solved numerically by the Runge-Kutta method of the fourth-order^50^. In the quantum approach, the time-dependent Schrödinger equation (4) is solved by the method of Chebyshev global propagation^51^. Wigner functions were calculated using an algorithm from the Ref.^52^, modified to allow efficient evaluation of Fourier transform only in a selected domain. All canonical oscillatory integrals were calculated using fortran cuspint library^53^. Additional details of the theoretical modeling can be found in the supplementary material.

## Results

### Classical dynamics

**Figure 1:**
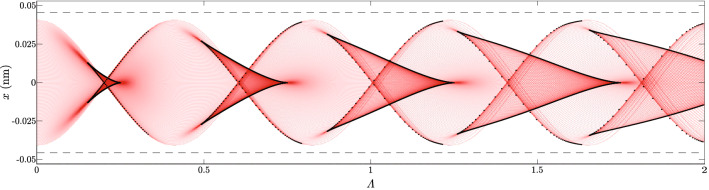
Family of classical proton trajectories corresponding to a parallel beam in the interval $0\le \Lambda \le 2$. Dotted black lines show caustic lines of the family. Thin dashed black lines show boundaries of the planar channel.

Figure 1 shows the obtained family of classical trajectories belonging to one unit cell of the potential *V*(*x*), together with associated caustic lines. Interestingly, the obtained family is morphologically identical with a trajectory family of protons transmitted through planar channels [110] of Si crystal. Morphological properties of the family and its relationship with the anharmonicity of the potential were investigated in detail in Ref.^48^. To avoid repetition, we will put emphasis only on features of the trajectory family related to structural stability.

As expected, the evolution of the proton beam is aperiodic. However, generated caustic lines are very regular. A repeating motif is a line resembling a bifurcation set of the $A_5$ catastrophe called the butterfly^26^. It consists of 4 branches combining pair-vise to form three cusps, two laying off, and the third laying on the channel axis. The proton density is largest at the apex of the cusp at the channel axis called a superfocusing point^48^. New superfocusing points appear periodically with period 0.5. The evolution of caustic lines progresses cyclically with each repetition called a rainbow cycle^54^.

**Figure 2:**
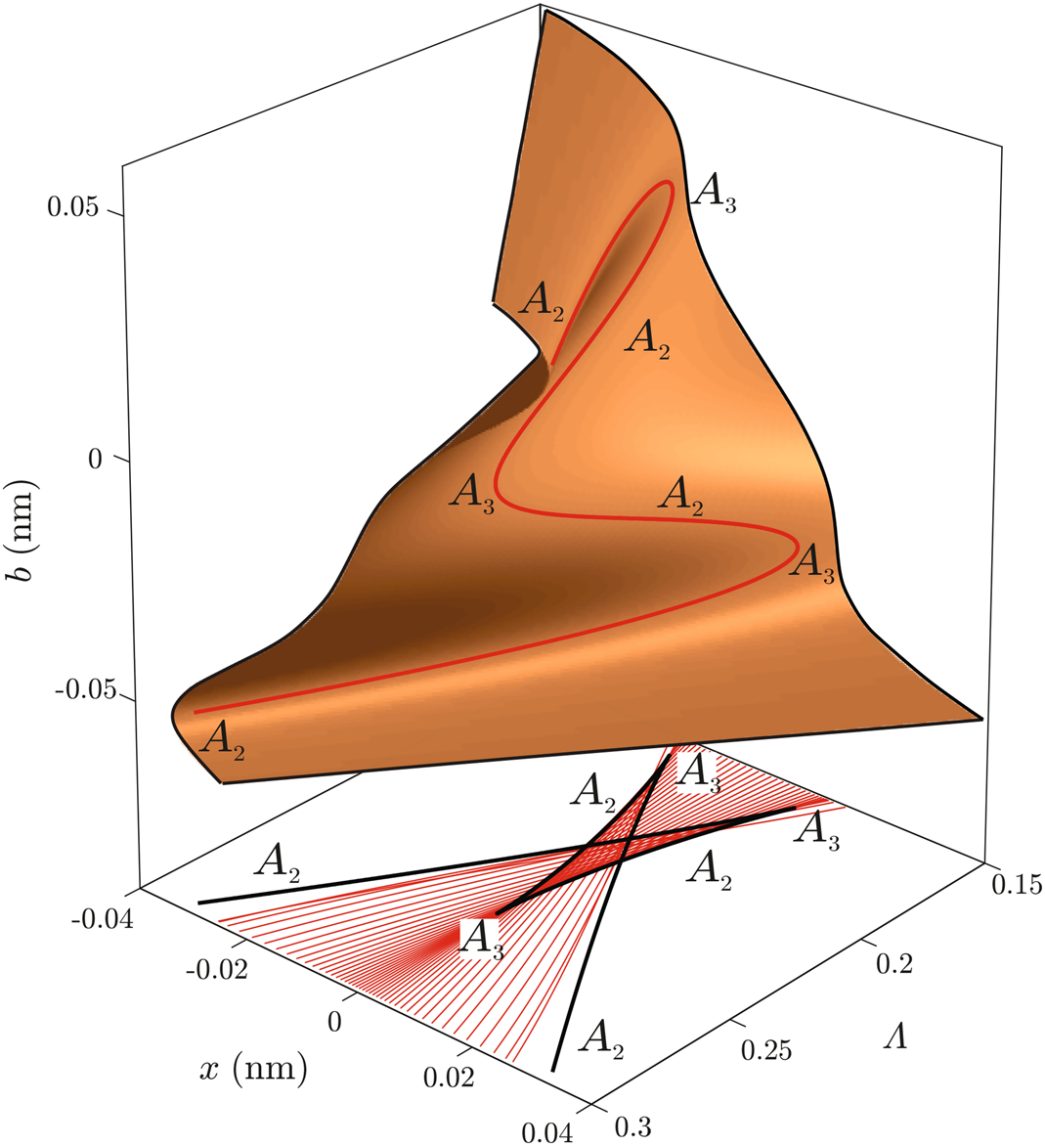
An equilibrium surface of the catastrophic model. The thick red line shows the critical set of the surface. The black line shows the projection of the critical line on the $(x,\Lambda )$ plane i.e. the caustic line of the catastrophic model. Thin red lines show the corresponding exact proton trajectories.

It is no coincidence that caustic lines resemble the shape of a certain catastrophic polynomial. This is a manifestation of Thom’s classification theorem which asserts that all structurally stable systems are isomorphic to some catastrophic prototype^25,27^. To demonstrate this fact, we have modeled caustic lines of the first rainbow cycle by the catastrophe $A_5$, which is the simplest catastrophe with the bifurcation set containing three cusps. The catastrophic model was constructed from a requirement that cusps of the catastrophic model match cusps of the caustic line. It was found that an appropriate model is given by the expression

17 $$\begin{aligned} A_5 = \frac{1}{6}\left( \frac{b}{b_0}\right) ^6+\frac{c_4}{4}\left( \frac{b}{b_0}\right) ^4+\frac{\Lambda -\lambda _0}{2\Lambda _0}\left( \frac{b}{b_0}\right) ^2+\frac{x}{x_0}\frac{b}{b_0}, \end{aligned}$$

were $c_4$ is the modulus of catastrophe and $\Lambda$ is the unfolding parameter. Details of catastrophic modeling are given in the supplementary material. To prove that catastrophe $A_5$ is structurally stable requires the use of topology and advanced mathematics. A much more accessible way to see that is by analysis of its equilibrium surface, defined by an equation $\frac{\text {d}}{\text {d}b}A_5 =0$, which is shown in Fig. 2. This equation defines implicitly family of functions $b\rightarrow x$ depending continuously on the parameter $\Lambda$. It can be understood as a series of model deflection functions glued together to form a surface. A red line in Fig. 2 shows critical points of the surface and represents a line along which the surface is folded. Its projection in the $(x,\Lambda)$ plane is the bifurcation set of the catastrophe $A_5$. Whenever an inward fold meets an outward fold they cancel each other out in the process equivalent to a saddle-node bifurcation. This process is equivalent to the $A_2$ catastrophic change of the family $\text {d}A_5/\text {d}b$, or to the $A_3$ *catastrophe* of the family $A_5$. Therefore, each fold of the surface represents graphically one $A_2$ catastrophe of the family $\text {d}A_5/\text {d}b$. Any deformation of the surface regardless of its size that preserves a folding structure gives a new surface topologically equivalent to the catastrophic prototype. To change the shape of the surface, new folds must be added or removed. This means that the morphology of the folded surface changes in an abrupt manner i.e. it is structurally stable.

### Quantum dynamics

Now when the classical limit is elaborated, we are ready to analyze additional structures arising from the self-interference of wave packets. For 2-MeV protons, the longitudinal wave vector is $k_z=0.21958$ fm${}^{-1}$. Since $k_zd_{111}=20 000\gg 1$ it is to be expected that a semiclassical approximation is applicable, and that approximate result should resemble the classical solution^7^. This concussion is consistent with Bohr–Lindhard’s condition^55, 56^ of applicability of the classical description of channeled particles $\kappa =Z_1Z_2m_pe^2/2\varepsilon _0\hbar ^2k_z=23.4164 \gg 1$. However, these heuristic arguments do not quantify to which extent classical features should be prominent.

We have analyzed the transmission of two proton beams, one having angular standard deviations $\Omega =0.1\Theta _c$, and the other having $\Omega =0.01\Theta _c$. Both divergences are small, thus respective ensembles represent quasi-parallel beams. Corresponding spatial standard deviations of wave packets are $\sigma _x=0.0038$ nm and $\sigma _x=0.0382$ nm, respectively. An interval containing a wave packet is approximately equal to $6\sigma _x$. In the case of the first ensemble, this interval is approximately $0.25 d_{111}$, while in the case of the second ensemble is approximately $2.5 d_{111}$. The first ensemble will be called a Gauss-like, while the second will be called a plane-wave-like ensemble. Note that according to the previous analysis both ensembles should behave identically.

**Figure 3:**
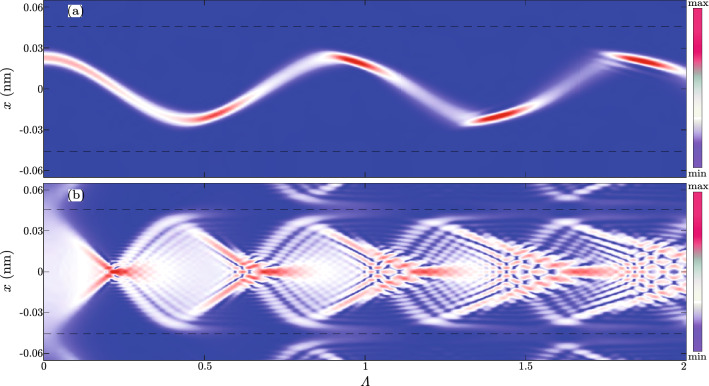
Evolution of the wave packet in the interval $0\le \Lambda \le 2$ of: **(a)** impact parameter $b=d_{111}/4$, and angular standard deviation $\sigma _\theta =0.1\Theta _c$; and **(b)** impact parameter $b=0$, and angular standard deviation $\sigma _\theta =0.01\Theta _c$. Horizontal lines show boundaries of the planar channel. Values of the probability density are represented by the color tone ranging from deepest blue to the deepest red.

Figure 3a shows an evolution of the wave packet from the Gauss-like ensemble having impact parameter $b=d_{111}/4$. Since the spatial divergence of the wave packet is small, tunneling of the proton wave function into neighboring potential wells is negligible. A self-interference of the wave packet is pronounced for $\Lambda >1.2$. The dominant maxima of the wave packet trace out a curve almost identical to the trajectory of the classical particle. The wave packet becomes the most focused at points of the maximal deflection of its *trajectory*.

Note that a quantum mean value of velocity must change sign near the point, denoted by ${\bar{\Lambda }}$, where trajectory deflects the most. This means that the quantum velocity field

18 $$\begin{aligned} v(x,\Lambda )=\frac{1}{|\psi (x,\Lambda )|^2}\frac{\hbar }{m_\text {p}}\mathfrak {I}\{\psi ^\dag (x,\Lambda )\nabla \psi (x,\Lambda )\}, \end{aligned}$$

have a nodal line somewhere in a rectangle of length $\Delta x$ and width $\Delta \Lambda$, centered at the point $(b,{\bar{\Lambda }})$. There function $v$ can be considered as small and approximated by $v(x,\Lambda )\approx u(\Lambda )\cdot x$. In that case, terms containing spatial derivatives of the probability density can be dropped from the equation of the continuity giving

19 $$\begin{aligned} |\psi (x,\Lambda )|^2\sim \exp \left[ -\frac{2\pi }{\omega }\int _{\Lambda _0}^\Lambda u(\lambda )\text {d}\lambda \right] \left| \psi \left( x,\Lambda _0\right) \right| ^2, \end{aligned}$$

where $\Lambda _0={\bar{\Lambda }}-\Delta \Lambda /2$. Note then in the vicinity of the first point of maximal deflection, the function $u(\Lambda )$ is negative for $\Lambda <{\bar{\Lambda }}$ while it is positive for $\Lambda >{\bar{\Lambda }}$. Upon entering the rectangle containing the nodal line, the shape of the wave packet remains unchanged. At first, its amplitude gets increased and its width decreased. After that, its amplitude gets decreased, and the wave packet gets wider. Therefore, the wave packet is the most focused approximately for $\Lambda ={\bar{\Lambda }}$. This is a manifestation of the wave packet accumulation effect, which was found to be important for an explanation of the quantum rainbow effect^35^.

Figure 3b shows the evolution of the wave packet from the Plane-wave-like ensemble having an impact parameter $b=0$. From the beginning, its self-interference is very pronounced. Although the width of the initial wave packet cowers three neighboring unit cells, tunneling between different cells is small. The classical caustic pattern from Fig. 1 is easily recognizable. The repeating pattern is similar to the butterfly canonical oscillatory integral $\chi _5$ [see Eq. (16)]. In contrast to the previous case, the amplitude of the wave packet is large near classical caustic lines. The wave packet is the most focused in the vicinity of classical superfocusing points. The probability density is again accumulated in the vicinity of nodal lines of the velocity field $v(x,\Lambda )$ which are very complicated in this case.

**Figure 4:**
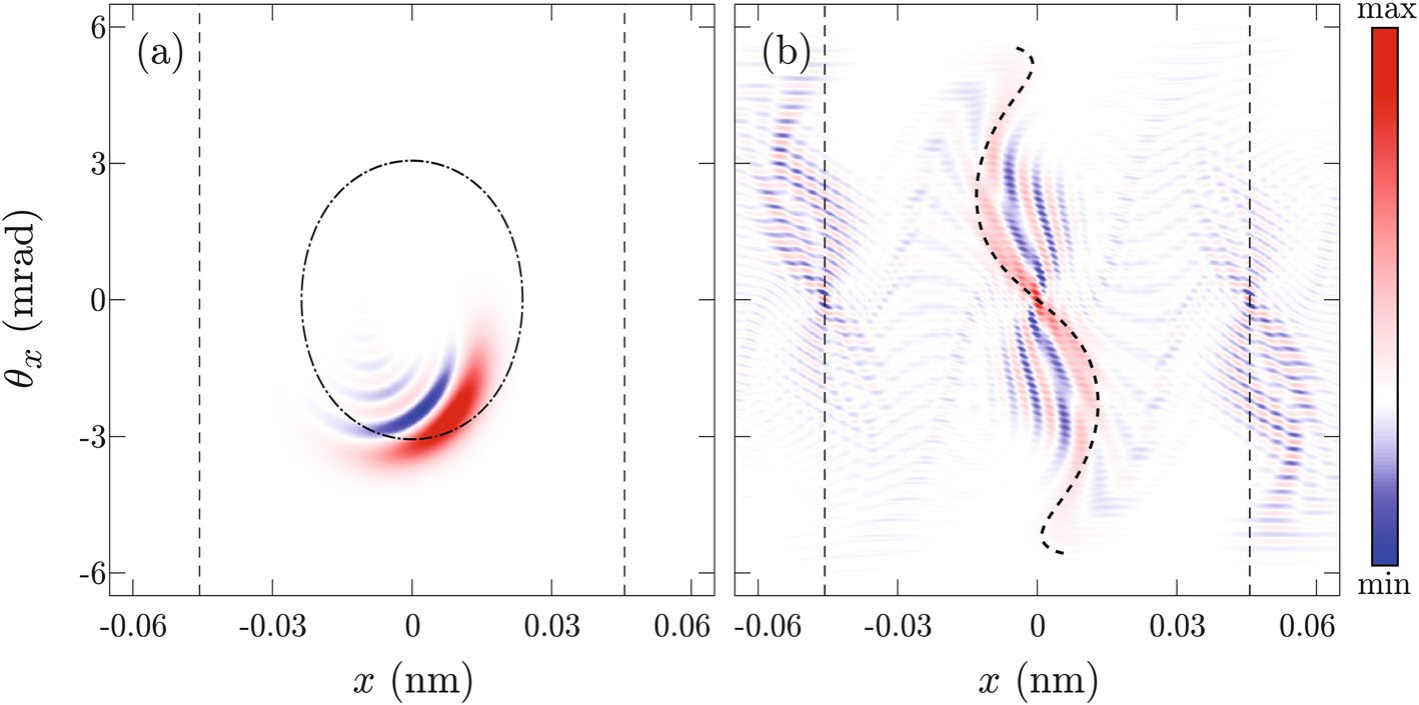
Wigner transformations of: **(a)** the wave function from Fig. 3a for $\Lambda =2$; **(b)** the wave function from Fig. 3b for the $\Lambda =0.6$. The thin dot-dashed black line shows the invariant torus corresponding to the state’s mean value of the transverse energy. The thick dashed black line shows the rainbow diagram, while thin dashed black lines show the boundaries of the planar channel. Values of the Wigner function are represented by the color tone ranging from the deepest blue to the deepest red.

A more instructive way to look at the proton dynamics is to inspect it in the phase space. For $\Lambda =2$, Wigner transformation of the wave packet from Fig. 3a is shown in Fig. 4a. The obtained Wigner function has a broad dynamical range. To highlight its shape a quantization function of its amplitude was taken to be $\text {erf}(aW)$. Here $\text {erf}$ stands for the error function^37^ while parameter $a$ was chosen that asserts amplification of small values and saturation of large values of the Wigner function The dot-dashed black line shows an invariant torus corresponding to the wave packets quantum mean value of the transverse energy

20 $$\begin{aligned} E_T(b,\sigma _x) = \frac{\hbar ^2}{8m_\text {p}\sigma _x^2}+\int \limits _{-\infty }^\infty V(x)|\psi _0(x)|^2\text {d}x. \end{aligned}$$

The wave packet from Fig. 3a makes more than two full revolutions tracing this curve (see video 1 of the supplemental material). Over time negative values of the Wigner function appear, and its shape starts resembling a fold diffraction catastrophe associated with the invariant torus^39^, modulated by a Gaussian.

Figure 4b shows the Wigner transformation of the wave packet from Fig. 3b for $\Lambda =0.6$. The quantization function was again taken to be $\text {erf}(aW)$. This time negative values of the Wigner function appear almost immediately. Note that the shape of the wave packet bears no relation to the invariant torus corresponding to its transverse energy $E_T$. In this case, the shape of the wave packet reflects the shape of the rainbow diagram ${\mathcal {R}}(\Lambda )$, which is in Fig. 4b shown by the thick dashed line (see also video 2 from the supplementary material). The resulting self-interference pattern can be described as a fold canonical diffraction pattern associated with the rainbow diagram^33,49^.

Note copies of the wave packet self-interference pattern, from the center of the unit cell, that are centered at classically unstable points $x=\pm d_{111}/2$, and called shadow copies^33^. These patterns do not have classical analogs and are very important for the understanding of the quantum entanglement^57,58^. However, they are nonclassical and unimportant for our analysis. Therefore, we shall not deal with them anymore.

**Figure 5:**
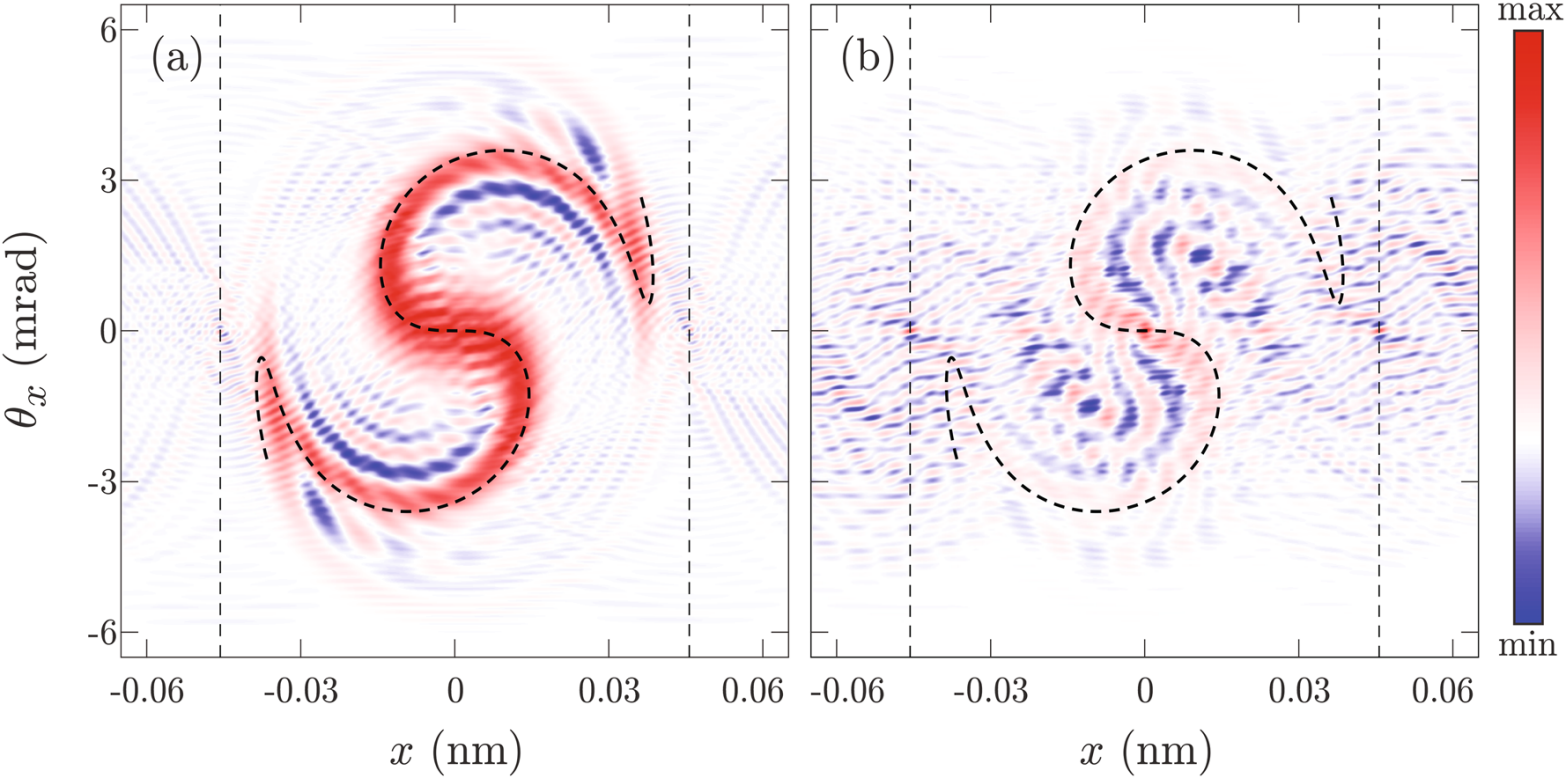
The Wigner functions of: **(a)** the Gaussian-like; and **(b)** the plane-wave-like ensembles for $\Lambda =2$, respectively. Thick dashed black lines show rainbow diagrams while thin dashed lines show boundaries of the planar channel. Values of the Wigner function are represented by the color tone ranging from the deepest blue to the deepest red.

For $\Lambda =2$ Wigner functions of Gauss-like and plane-wave-like ensembles, obtained in accordance with Eq. (14), are shown in Fig. 5a, and b, respectively Corresponding rainbow diagrams are shown by thick black dashed lines. The first thing to observe is that both Wigner functions reflect the shape of the rainbow diagram. This is not surprising since the classical limit of both Wigner functions is precisely the rainbow diagram^33,43^. Positive values of the Wigner function of the Gaussian-like ensemble are much more concentrated near the rainbow diagram than in the case of the plane-wave-like ensemble. The self-interference pattern from Fig. 5a is much more regular than the corresponding self-interference pattern from Fig. 5b. Note that the smallest structures in Fig. 5a and b have approximately the same size. This may seem surprising because there is a considerable difference between the initial angular divergence of Gauss-like and plane-wave-like ensemble. It is tempting to make a false conclusion that the Wigner function from Fig. 5a should be a *blurred* version of the Wigner function from Fig. 5b. Actually, filtering only reduces image contrast or sharpness of structures forming the Wigner function landscape, it does not remove them completely (see supplementary material). The non-uniform quantization used in Fig. 5 provides sufficient dynamical range making all relevant structures visible. The size of the smallest self-interference structures is related to the size of the Wigner function support^33,59^ which is equal in both cases.

To quantify a degree of the self-interference present in an ensemble we shall calculate how much of the Wigner function norm is contained in the negative part of its spectrum. Our investigation will be focused on an area $[-\frac{d_{111}}{2},\frac{d_{111}}{2}]\times [-\Theta _c,\Theta _c]$ of the phase space containing the classical system. Any features outside of it clearly do not have classical analog and shall be neglected. We shall define an auxiliary Wigner function ${\overline{W}}$ by the relation

21 $$\begin{aligned}{\overline{W}}(x,\theta _x)=\left\{ \begin{array}{llll}-W(x,\theta _x),&{}\hbox {for } |x|\le \frac{ d_{111}}{2},|\theta _x|\le \Theta _c, \hbox { and }W(x,\theta _x)<0,\\ 0,&{}\hbox {othewise},\end{array}\right. \end{aligned}$$

and calculate its mean value in the examined region

22 $$\begin{aligned} \eta = \frac{1}{2d_{111}\Theta _c}\int \limits _{-d_{111}/2}^{d_{111}/2}\int \limits _{-\Theta _c}^{\Theta _c}{\overline{W}}(x,\theta _x)\text {d}x\text {d}\theta _x, \end{aligned}$$

which will be called negativity. Note that this criterion takes into the account size of the domain where the Wigner function is negative as well as its magnitude. Therefore, it can be used for comparison between levels of the self-interference of two different ensembles. For classical ensemble $\eta =0$, therefore, negativity can also serve as an indicator of the classicality. In the case of the Gauss-like ensemble $\eta =0.0677$ (nm$\cdot$mrad)${}^{-1}$, while for the plane-wave-like ensemble $\eta =0.4856$ (nm$\cdot$mrad)${}^{-1}$. Since obtained negativities are significantly different, we can claim that the Gauss-like ensemble is more classical.

## Discussion

One way to understand why one ensemble seems to be more classical than the other is to assume that obtained exact wave packets are significantly different from their corresponding semiclassical approximations. If this assumption is correct then different behavior of ensembles is merely a consequence of the different behavior of their respective members. This may be caused by some subtle reason not captured by heuristic arguments of the applicability of the semiclassical analysis.

**Figure 6:**
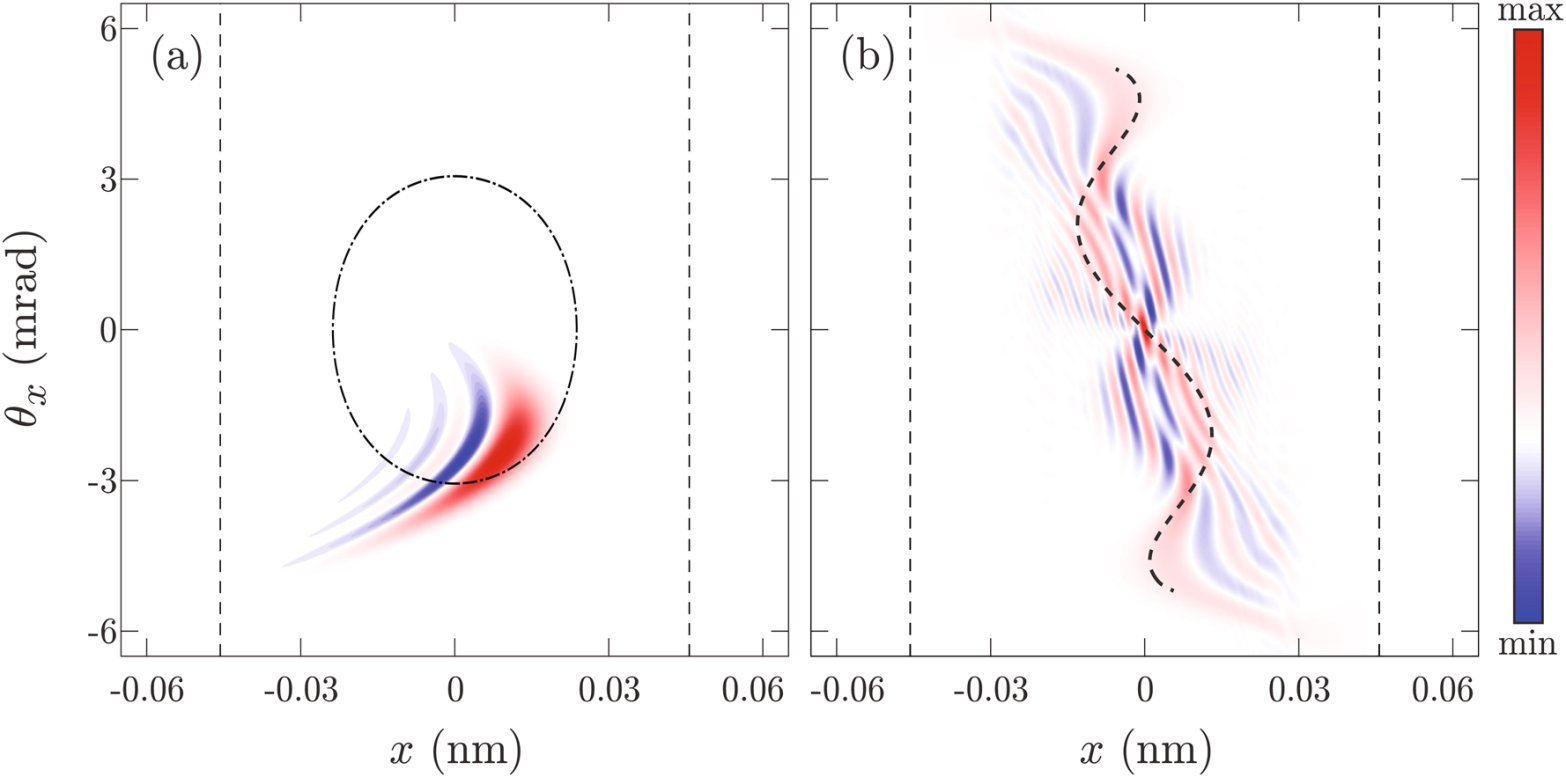
Semiclassical approximation of Wigner functions form: **(a)** Fig. 4a; and **(b)** Fig. 4b, respectively. The thin dot-dashed black line shows the invariant torus corresponding to the state’s mean value of the energy. The thick dashed black line shows the rainbow diagram, while thin dashed black lines show the boundaries of the planar channel. Values of the Wigner function are represented by the color tone ranging from the deepest blue to the deepest red .

To investigate this possibility we have used classical spatial and angular deflection functions to calculate semiclassical approximations of exact Wigner functions from Fig. 4. The semiclassical approximation of the Wigner function from Fig. 4a, given by the relation

23 $$\begin{aligned} \begin{array}{ll} W_b(x,\theta _x)=&{}\frac{\gamma _1}{\sqrt{2\pi \sigma _x^2}}\exp \left[ \frac{1}{\gamma _2}\left( \frac{1}{12\gamma _2k_z^2\sigma _x^2} - (p_1x+r_1) - \frac{r_2}{p_1}(\theta _x-p_0)\right) \right] \\ &{}\times \,\chi _2\left[ \gamma _1\left( \frac{1}{8\gamma _2k_z^2\sigma _x^2} - (p_1x+r_1) + \frac{r_2}{p_1}(p_0 - \theta _x) + \frac{r_3}{p_1^2}(p_0 - \theta _x)^2\right) \right] , \end{array} \end{aligned}$$

is shown in Fig. 6a. Here $\gamma _1=\root 3 \of {4k_z^2/r_3}$, and $\gamma _2=2r_3\sigma _x^2$ are dimensionless parameters, while $\chi _2$ is Airy’s function of the first kind which. The semiclassical approximation of the Wigner function from Fig. 4b, given by the expression

24 $$\begin{aligned} W_b(x,\theta _x) = - \frac{\zeta }{2\pi d_{111}}\chi _4\left[ \zeta \left( p_1x+r_2\frac{\theta _x}{p_1}+r_4\frac{\theta _x^3}{p_1^3}+r_6\frac{\theta _x^5}{p_1^5}\right) ,0,\zeta ^3\left( \frac{3}{4}r_4\frac{\theta _x}{p_1}+\frac{5}{2}r_6\frac{\theta _x^3}{p_1^3}\right) \right] , \end{aligned}$$

is shown in Fig. 6b. Here $\zeta =\root 5 \of {16k_z^4p_1/5s_6\theta _x}$ is an auxiliary function, while $\chi _4$ is the swallowtail diffraction pattern. Details of the semiclassical modeling can be found in the supplementary material. Figure 6 shows that semiclassical results, given by structurally stable canonical diffraction patterns, correctly reproduce all essential features of exact solutions from Fig. 4. Therefore, both semiclassical results are equally accurate.

Another way to understand the observed behavior is to assume that angular divergences strongly influences the ability of wave packet for self-interference. To investigate this possibility we have analyzed the correspondence limit. An arbitrary quantum state can be represented as a superposition of normalized energy eigenvectors $\psi _n$

25 $$\begin{aligned} \psi _b(x,\Lambda ) = \sum _n c_n(b)\exp \left[ -2\pi i\Lambda \frac{E_n}{\hbar \omega }\right] \psi _n(x), \end{aligned}$$

where $|c_n|^2$ ($\sum _n|c_n|^2=1$) is a statistical weight of the eigenstate state $\psi _n$, while $E_n$ is corresponding $n$-th energy eigenvalue. State $\psi _n$ has $n$ nodes in the interval of the unit cell. If this state is excited, then over time, distribution $|\psi _b(x)|^2$ will develop structures of size $d_{111}/(n + 1)$^59^. Note that rainbow peaks of the classical distribution are very sharp^48^. To reproduce such structures, states of large quantum numbers must be excited.

**Figure 7:**
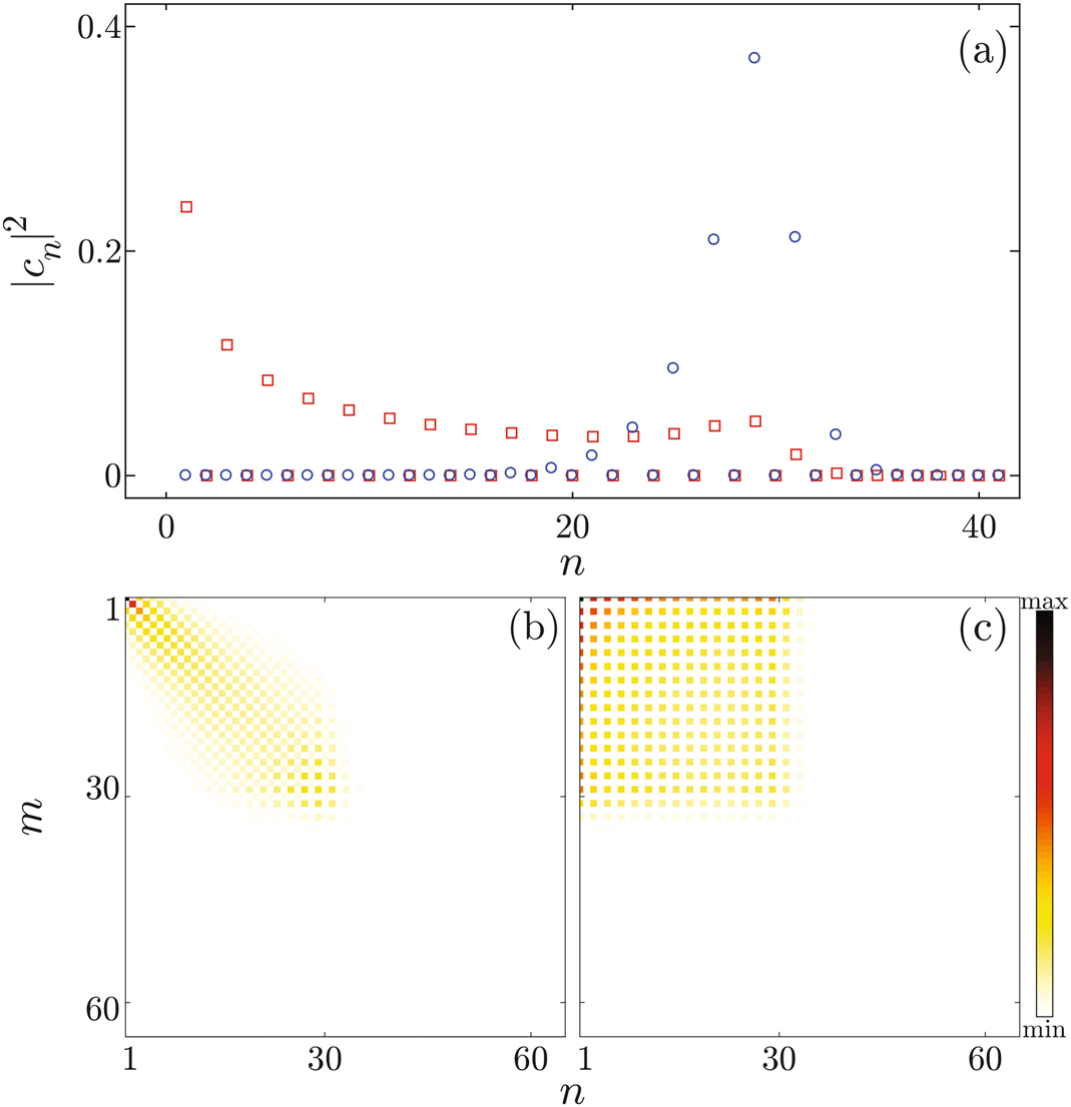
**(a)** Magnitudes squared of expansion coefficients for states from the Figs. 3a and b shown by the blue circle and the red square markers, respectively. Modulus of the density matrix elements in the energy eigenbasis $\psi _n$ for: **(b)** Gauss-like and **(c)** plane-wave-like ensembles. Values of the matrix elements are represented by the color tone ranging from white to the deepest red .

Figure 7a shows non-negligible weights of states from Fig. 3, obtained numerically. Blue circular markers show weights of the Gauss-like wave packet, while red square markers show weights of the plane-wave-like wave packet. In the case of the Gauss-like state, the distribution of weights is approximately Gaussian, while in the case of the plane-wave-like state, weights are distributed much more uniformly. This difference is unimportant since it only indicates that the wave packet from the plane-wave-like ensemble covers the whole unit cell. In both cases only 36 lowest states are excited.

To estimate an ability for the self-interference of an ensemble we have inspected the density matrix operator which is, in the basis $\psi _n$, represented by the following matrix

26 $$\begin{aligned} \rho _{mn}(\Lambda )=\int \limits _{-\infty }^{\infty }\psi _m^\dag \hat{\rho }(\Lambda ) \psi _n\text {d}x=\sum _{b}p_{b} c_m^\dag (b)c_n(b)\exp \left[ 2\pi i\Lambda \frac{E_n-E_m}{\hbar \omega }\right] . \end{aligned}$$

Note that $|\rho _{mn}(\Lambda )|=|\rho _{mn}(0)|$. Figure 7b, c show matrices $|\rho _{mn}|$ of the Gauss-like and the plane-wave-like ensemble, respectively. It is interesting that in both cases the largest submatrix with non-negligible elements is of dimension $36\times 36$. This means that all states from both ensembles belong to the same subspace spanned by eigenvectors shown in Fig. 7a. A density matrix of the Gauss-like ensemble is diagonally dominant, while in the case of plane-wave-like ensemble values of non-zero elements are practically uniformly distributed. Distributions of the matrix elements reflect distributions of expansion coefficients shown in Fig. 7a. Therefore, we may conclude that both ensembles have roughly equal ability to produce classical structures.

**Figure 8:**
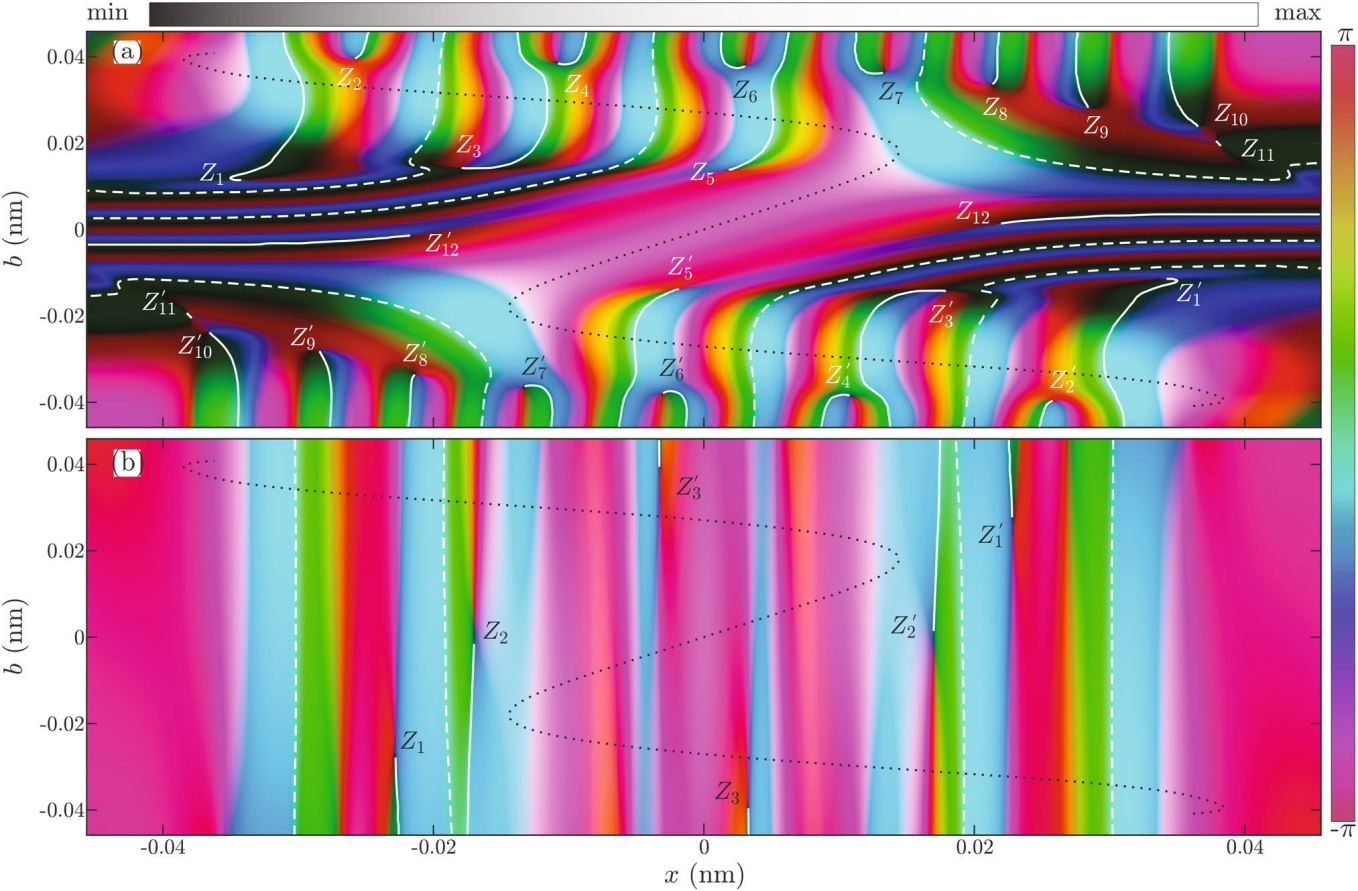
Domain color representation of the complex function $\psi (x,b)$ of the **(a)** Gauss-like; and **(b)** plane-wave-like ensembles, for $\Lambda =2$. Amplitude $|\psi (x,b)|$ is shown by the color lightness while phase $\arg {\psi (x,b)}$ is shown by the color tone. Full and dashed white lines show branch-cuts of the function $\arg {\psi (x,b)}$. Zeros of the function $\psi (x,b)$ are marked by letters. Doted black lines show the classical deflection function $X(b)$ .

Preceding analysis shows that the self-interference of wave packets from both ensembles can produce patterns of approximately equal level of detail. However, there is no guarantee that their distribution will produce classical structures. The appearance of such structures would depend on a degree of coordination between the self-interference of different wave packets. This requires inspection how ensemble wave functions $\psi (x,b)$ depends on the impact parameter $b$. For $\Lambda =2$ domain color representation of the complex valued functions $\psi (x,b)$ of the Gauss-like and the plane-wave-like ensembles are shown in Fig. 8. In this representation, a color is assigned to each point of the $(x,b)$ plane. The phase of a complex number is represented by color hue following the color wheel, while the magnitude of the complex number is indicated by color lightness. Because of inversion symmetry of the potential $V(x)=V(-x)$, the complex function $\psi (x,b)$ is invariant on the transformation $\psi (x,b)=\psi ^\dag (-x,-b)$, and obtained image is centrally symmetric.

An eye of the beholder is immediately drawn to discontinuous jumps in the coloring of images, marked by white lines. They represent jumps of $2\pi$ radians in the phase of the complex functions. Jumps marked by dashed white lines are removable. A shift of $2\pi$ applied in the appropriate area (whose boundary is the dashed line) can make a local coloring of the image continuous again.

The situation is different with discontinuities marked by full white lines. They emerge out of the points where all colors meet and color lighting is the lowest. This singularity occurs whenever the magnitude of the complex function is zero and its phase is indeterminate. In the complex analysis, the full white lines are known as branch cuts of the complex function and they mark locations where different branches of the phase function meet^60^. Almost identical structures occur in the propagation of waves where they are known as phase dislocations^61,62^.

In the case of the Gauss-like ensemble from Fig. 8a, there are 24 zeros. Irreducible zeros are labeled $Z_1,\ldots , Z_{12}$ while their symmetrical equivalents are denoted by the same symbol additionally marked by prime. In the case of the plane-wave-like ensemble, shown in Fig. 8b, there are only 6 zeros. Irreducible zeros are labeled by $Z_1, Z_2, Z_{3}$, while their symmetrical equivalents are again denoted by the same symbol marked by prime. Note vertical stripes of colors running almost parallel to the branch cuts and of alternating high and low lighting. This indicates the existence of the amplitude-phase locking between different members of the ensembles.

Corresponding classical deflection function $X(b)$ is in Fig. 8a,b shown by dashed black lines. This function has four critical points i.e. it shows four rainbow points. In both cases, the highest color lightnesses are to be found in the vicinity of the rainbow points. In Fig. 8a ridges of high lightness are formed around the deflection function. In the case of the amplitude function from Fig. 8b, the situation is different. Dependence of the function $\psi (x,b)$ on the parameter $b$ is weak, and it is difficult to recognize any structures reflecting the shape of the function $X(b)$. Here probability densities of almost all wave packets are large in the vicinity of four rainbow points (see also Fig. 3b). Therefore, both ensembles reflect the shape of the classical caustic pattern in two different ways.

The significance of zeros for dynamics of the ensemble is best appreciated by inspection of the equation of continuity

27 $$\begin{aligned} m_\text {p}\partial _t\rho +\rho \cdot \partial _{xx}^2 S+\partial _x\rho \cdot \partial _x S=0, \end{aligned}$$

where $\psi =\sqrt{\rho }\exp [iS/\hbar ]$. At the zero $(x_Z, b_Z)$, probability density is $\rho (x_Z, b_Z)=0$. Since it is always $\rho \ge 0$, the zero-point must also be a local minimum forcing $\partial _x\rho (x_Z, b_Z)=0$. The equation of continuity (27) then implies that $\partial _t\rho (x_Z, b_Z) =0$. Continuous dependence of the function $\psi (x,b)$ on the variable $b$, implies existence of a region in the $(x,b)$ space in which $\partial _t\rho (x,b)\approx 0$, which will be called a domain of zero. In the domain of zero, except at the point $(x_Z, b_Z)$, it generally holds $\rho (x, b)\ne 0$. Again because of the continuous dependence on the variable $b$, in the domain of zero minima $\partial _x\rho (x,b)=0$ still exist and force $\partial _{xx}^2 S(x,b)=0$ at same locations. In the area between two neighboring domains of zero in the $x$ direction, it is also $\partial _t\rho (x, b) \approx 0$, since it is negligible in the neighboring areas. Because of continuity, there are points where density $\rho$ has local maxima. By the same argumentation $\partial _{xx}^2 S(x,b)=0$ at those points. The established connection between critical points of the amplitude function and degenerate critical points of the phase function is similar to a relationship between dislocations and saddle points of 2d waves^63^.

If the dependence on the variable $b$ is weak then all extrema will be perfectly aligned and their contributions will be locally amplified. Additionally, if domains of zero cower the whole $(x,b)$ space then the self-interference is globally coordinated, and quantum interference effects should be very pronounced in the ensemble. This is a precise formulation of the coordinated self-interference introduced to explain the quantum rainbow effect^35^. To show it explicitly we have calculated trajectories of the points satisfying the equation $\partial ^2_{xx}S=0$ and plot it together with the probability density $\rho (x,b)$.

**Figure 9:**
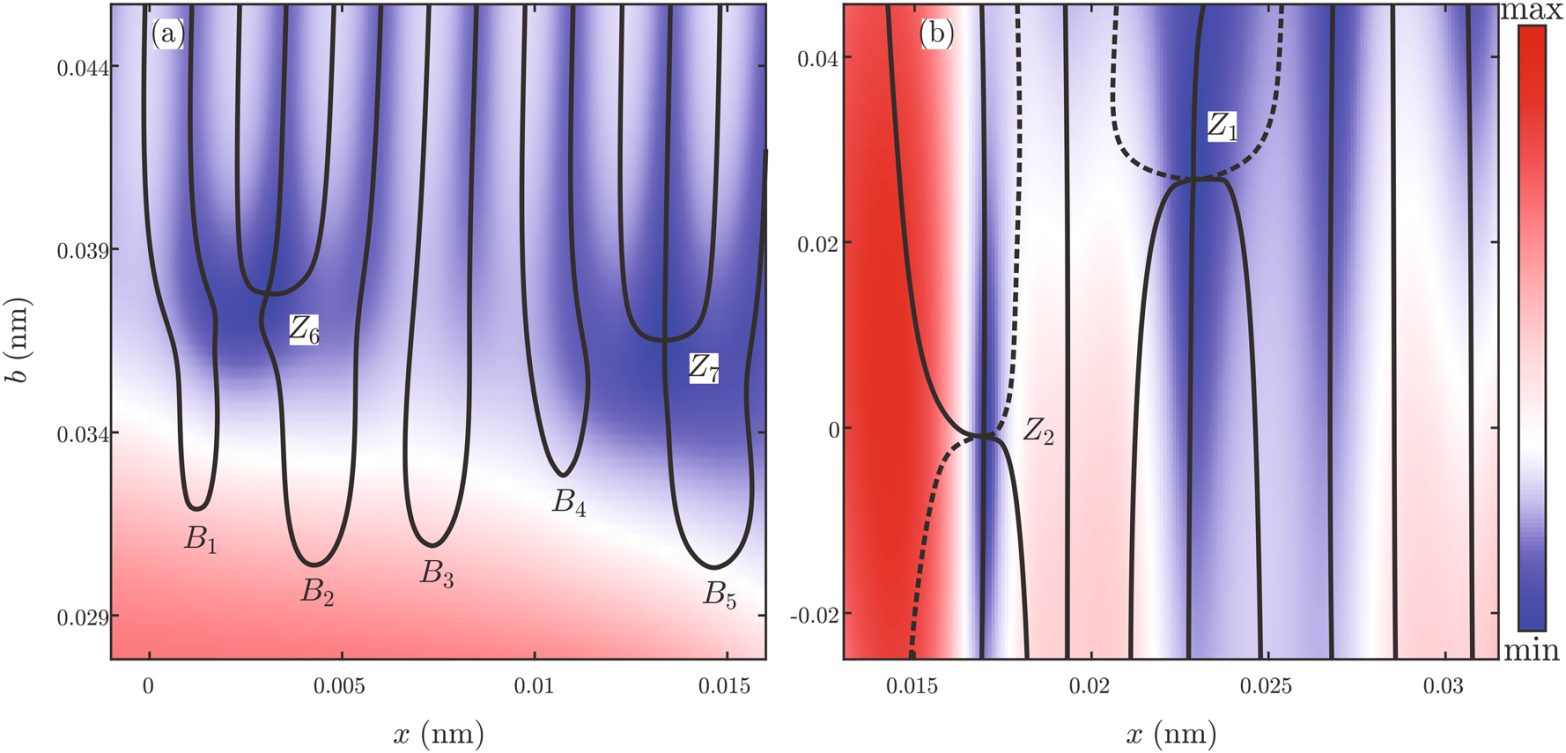
Enlarged views of the probability densities $\rho (x,b)$ and trajectories of points $\partial ^2_{xx} S(x,b)=0$ for $\Lambda =2$ in the case of: **(a)** Gauss-like and **(b)** plane-wave-like ensemble. Full and dashed black lines correspond to inflection and undulation points, respectively of the function $S$. Values of the function $\rho$ are represented by the color tone ranging from the deepest blue to the deepest red.

For $\Lambda =2$ enlarged views of the probability density $\rho (x,b)$ near zeros of the Gauss-like and the plane-wave-like ensemble are shown in Fig. 9a,b, respectively. Associated trajectories of inflection and undulation points are shown by full and dashed black lines respectively. Figure 9a shows that extrema of the probability density are perfectly aligned with inflection points (places where $S\sim x^3\pm ax$) of the phase function only in the domain of zero. Outside of it, the correlation between them is very weak. Some inflections emerge out of points labeled $B_1,\ldots ,B_5$, while others emerge out of zeros $Z_6$ and $Z_7$. This is understandable since according to the singularity theory the number of real critical points of the function $\partial _x S$ can change by 2. A sudden appearance of the critical point pair is a process known as the saddle-node bifurcation, which is fully equivalent to the behavior of critical points of the $A_2$ catastrophe. Another elementary process known in the bifurcation theory as the pitch-fork bifurcation is splitting of one critical point into three. This process happens at the zeros $Z_6$ and $Z_7$ and is equivalent to the behavior of critical points of the $A_3$ catastrophe. Figure 9b displays identical behavior of the plane-wave-like ensemble. Extrema of the functions $\rho$ are perfectly aligned with the trajectories of the inflection and undulation points of the phase function (places where $S\sim \pm x^4\mp ax$). In this case, reconfiguration of critical points of the function $\partial _x S$ happens at zeros $Z_1$, $Z_2$, through a more complicated process consisting of two back-to-back pitch-fork bifurcations. Inspection of this behavior in the vicinity of all other zeros confirmed that pitch-fork bifurcations are connected with the zeros of the $\partial ^2_{xx}S$.

Now we can explain the difference between the behavior of the Gauss and plane-wave-like ensembles. In the case of the Gauss-like ensemble, domains of zero are only regions where wave packets self-interfere in a coordinated way. Note that domains of zeros $Z_1,\ldots , Z_{12}$ practically cower the whole $x$-axis, and are not aligned with their symmetrical images $Z_1',\ldots , Z_{12}'$. As a result self-interference of wave packets is suppressed in the ensemble making the classical underlying rainbow diagram more visible in Fig. 5a. In the case of the plane-wave-like ensemble, the weak dependence on the impact parameter force all members of the ensemble to behave essentially in the same manner. Domains of zeros break initial global coordination by the introduction of the local domains of more strongly correlated behavior. Note that domains of zeros $Z_1$, $Z_2$, and $Z_3$ cover almost the entire $b$-axis. Their projections onto the $x$-axis do not overlap with the projection of their symmetrical images $Z_1'$, $Z_2'$, and $Z_3'$. Since the number of domains of zero is small, and they are narrow and long, the level of coordination between different members of the ensemble remains large. This explains why the Wigner function from Fig. 5b shows a more pronounced self-interference.

## Conclusions

We have analyzed the transmission of the proton beam through the [111] planar channels of the tungsten crystal of small and negligible angular divergence. The main focus of the analysis was on the ability of ensembles to produce classical structures.

It was found that the characteristic feature of the classical limit is a repeated appearance of the structurally stable caustic lines representing the underlying framework onto which quantum effects are superimposed. Both ensembles were found to have roughly equal capacity for self-interference. Wigner transformations of wave packets from both ensembles were successfully modeled by the canonical diffraction patterns, indicating that quantum self-interference is also structurally stable.

Examination of ensemble wave function $\psi (x,b)$, reveled the number of branch cuts emanating out the phase singularities i.e places where magnitude of the function $\psi (x,b)$ is zero. Through branch cuts its influence is extended to the wave packets of neighboring impact parameters forming regions in the $(x,b)$ space of the strongly coordinated behavior called domains of zero. Interlocking of the nearest domains leads to perfect alignment of the extrema of the amplitude function $|\psi (x,b)|$ and inflexion and undulation points of the phase function $\arg \{\psi (x,b)\}$. This is the underlying topological mechanism that produces coordinated self-interference of wave packets. All observed features are a consequence of continuous dependence of the function $\psi (x,b)$ on the variable $b$, and the fact that members of the structurally stable family of functions can be partitioned into topologically equivalent classes^25,27^.

We have shown that arrangement of the domains of zero is responsible for amplification or suppression of the wave packet self-interference in the ensemble. Therefore, singularities of the complex function $\psi (x,b)$ influence the level of the *classicality* of the quantum structurally stable ensemble. For this kind of systems, the classical level of reality emerges out the underlying quantum dynamics if contributions of different domains of zero suppress each other in the ensemble. The quantum-to-classical transition happens on the level of the ensemble without the need for quantum dynamics of wave packets to become classical. Such behavior represents an example of the deducible emergence property^64^. Our work also shows that the phase function of the ensemble $\arg \{\psi (x,b)\}$ is over-determined. It contains information about locations of extrema of the corresponding amplitude function $|\psi (x,b)|$. In our future publications, we will treat $\arg \{\psi (x,b)\}$ as Riemannian surface and try to relate the dynamics of the quantum system to changes in its geometry. We will investigate whether is possible to deduce relative relationship between extreme values of the function $|\psi (x,b)|$ out of the function $\arg \{\psi (x,b)\}$.

## Supplementary information

Supplementary Information.

Supplementary Video 1.

Supplementary Video 2.
