# Supplementary material for: Coordinated self-interference of wave packets: a new route towards classicality for structurally stable systems
Source: Sci Rep. 2020 Oct 12;10:16949. doi: 10.1038/s41598-020-72965-w (PMC7550619; doi:10.1038/s41598-020-72965-w)
Supplement: Supplementary file 1 — Supplementary Information. [file 41598_2020_72965_MOESM1_ESM.pdf]

Coordinated self-interference of wave packets: A new route towards classicality for structurally stable systems

M. Ćosić* and S. Petrović

Laboratory of Physics, Vinča Institute of Nuclear Sciences, University of Belgrade, P. O. Box 522, 11001 Belgrade, Serbia

S. Bellucci

INFN - Laboratori Nazionali di Frascati, 1-00044, Frascati (Rome), Italy

(Dated: September 4, 2020)

* **Corresponding author:** mcosic@vinca.rs

SUPPLEMENTARY MATERIAL

A. Theoretical framework

Let a_L, and N_a denote lattice constant and a number of atoms contained in the BCC unit cell of the tungsten crystal. Spacing between [111] planes is $d_{111} = \sqrt{3}a_L/6$, while corresponding surface density is $\sigma_{111} = N_a d_{111}/a_L^3$.

We assume that $y0z$ plane of the Cartesian coordinate system is parallel to [111] planes of the tungsten crystal with x axis orthogonal to them. Quasi-parallel proton beam of kinetic energy $E_k = 2\text{MeV}$ was assumed to be aligned with the z axis of the coordinate system.

Let $\boldsymbol{r} = (x, y, z)$ denotes a proton-tungsten separation vector. To construct proton-crystal interaction potential we start from the Molieré's approximation of the Thomas-Fermi's proton-tungsten interaction potential [1, 2]

$$U(\boldsymbol{r}) = \frac{Z_1 Z_2 e^2}{4\pi\varepsilon_0|\boldsymbol{r}|} \sum_{n=1}^{3} \alpha_n \exp\left[-\beta_n \frac{|\boldsymbol{r}|}{a_\mathrm{s}}\right], \tag{1}$$

where $Z_1 = 1$, and $Z_2 = 74$ are proton and tungsten atomic numbers, e is elementary charge, ε_0 is vacuum permittivity, and $\boldsymbol{\alpha} = (0.35, 0.55, 0.1)$ and $\boldsymbol{\beta} = (0.3, 1.2, 6)$ are dimensionless Molière's fitting parameters. In the Thomas-Fermi's model screening length is defined by an expression $a_\mathrm{s} = \frac{\varepsilon_0\hbar^2}{m_\mathrm{e}e^2}\sqrt[3]{\frac{9\pi^5}{2Z_2}}$ where m_e is electron mass, and $\hbar$ is reduced Planck's constant. Proton-tungsten interaction potential (1) should be averaged over distribution of atom displacements from their equilibrium positions $\boldsymbol{u}$ given by the expression

$$P_\mathrm{th}(\boldsymbol{u}) = \frac{1}{(2\pi\sigma_\mathrm{th}^2)^{3/2}} \exp\left[-\frac{|\boldsymbol{u}|^2}{2\sigma_\mathrm{th}^2}\right], \tag{2}$$

with a standard deviation given by the following relation

$$\sigma_\mathrm{th}^2 = \frac{3\hbar^2}{A_\mathrm{r} m_\mathrm{u} k_\mathrm{B} \Theta_\mathrm{D}} \left(\frac{\Theta_\mathrm{D}}{T} D_\mathrm{f}(T/\Theta_\mathrm{D}) + \frac{1}{4}\right), \tag{3}$$

here $A_\mathrm{r} = 183.85$ is tungsten atomic weight, $m_\mathrm{u} = 1.6605 \cdot 10^{-27}$ kg is universal atomic mass unit, $\Theta_\mathrm{D} = 310$ K is the tungsten Debye temperature [2], $k_\mathrm{B} = 1.3806 \cdot 10^{-23}$ J/K is Boltzmann's constant, T is sample's absolute temperature, and D_f is the Debye's function. Since scattering angles of channeled particles are small, the continuous approximation could be used. The resulting continuous planar-averaged potential is given by multiple integrals

$$V_{111}^\mathrm{th}(x) = \sigma_{111} \int\limits_y \int\limits_z \int\limits_{\boldsymbol{u}} U(\boldsymbol{r} - \boldsymbol{u}) P_\mathrm{th}(\boldsymbol{u}) \mathrm{d}^3\boldsymbol{u} \mathrm{d}y \mathrm{d}z, \tag{4}$$

For the Molière's potential integrals (4) can be evaluated analytically giving

$$V_{111}^{\rm th}\,(\,x) = \frac{Z_2 e^2 a_{\rm s}}{4\sqrt{2}\epsilon_0 d_{111}^2}\sum_{n=1}^{3}\frac{\alpha_n}{\beta_n}\exp\left[\frac{\beta_n^2\sigma_{\rm th}^2}{2a_{\rm s}^2}\right] \times\left\{\exp\left[-\frac{\beta_n|x|}{a_{\rm s}}\right]\mathrm{erfc}\left(\frac{\beta_n\sigma_{\rm th}}{\sqrt{2}a_{\rm s}}-\frac{|x|}{\sqrt{2}\sigma_{\rm th}}\right)+\exp\left[\frac{\beta_n|x|}{a_{\rm s}}\right]\mathrm{erfc}\left(\frac{\beta_n\sigma_{\rm th}}{\sqrt{2}a_{\rm s}}+\frac{|x|}{\sqrt{2}\sigma_{\rm th}}\right)\right\}, \tag{5}$$

here erfc is complementary error function [3]. In the final step potential of the planar channel was expressed as a sum of contributions of individual atomic planes. In the coordinate system attached to the midpoint between two nearest planes, the arrangement of planes can be viewed as a layered structure with each layer comprised of two plains. Planes of the m-th layer are to be found at distances $d_m = (m+\frac{1}{2})d_{111}$ from the coordinate origin. They enclose all planes of the layer order less than m. The channel potential is a periodic function with period d_{111} given by the following expression

$$V(x) = \sum_{m=0}^{N}\left(V_{111}^{\rm th}(x+d_m) + V_{111}^{\rm th}(d_m - x)\right) - V_0. \tag{6}$$

Each term of the sum gives a contribution of one layer which is in its own right a sum of contributions coming from the left and the right plane, respectively. Constant $V_0 = 2\sum_m V_{111}^{\rm th}(d_m)$ was introduced in order to have $V(0) = 0$. In principle number of terms in the sum should be infinite, however since potential (5) is a rapidly decreasing function, sum (6) can be truncated after N-th term.

In channeling it is usually $E_k \gg V(d_{111}/2)$. As a consequence maximal deflection angle, a classical particle could have while still being channeled, is given by the expression

$$\Theta_c = \sqrt{\frac{V(d_{111}/2)}{E_k}}, \tag{7}$$

which is called a critical channeling angle. For thin crystals, considered here, energy loss and fluctuation of the scattering angle can be neglected. Newton's equations governing proton dynamics are

$$m_{\rm p}\frac{\mathrm{d}^2\boldsymbol{r}}{\mathrm{d}t^2} = -\nabla V(x), \tag{8}$$

where $m_{\rm p}$ is proton mass, while t denotes the time. This means that motion in y and z directions is given by equations

$$y = const, \quad z = \sqrt{2m_{\rm p}E_k}t. \tag{9}$$

Since dynamics in the direction parallel to crystal planes is trivial it will be disregarded, and the problem treated as essentially one-dimensional.

In the transverse direction, proton dynamics is governed by Hamilton's equations of motion

$$\frac{\mathrm{d}}{\mathrm{d}t}\theta_x = -\frac{\partial_x V(x)}{\sqrt{2m_\mathrm{p}E_k}}, \quad \frac{\mathrm{d}}{\mathrm{d}t}x = \sqrt{2\frac{E_k}{m_\mathrm{p}}}\theta_x. \tag{10}$$

In order to represent parallel beam initial conditions should be in form $\theta_x(t=0)=0$, and $x(t=0)=b$, with uniform distribution of the proton impact parameter b. Trajectories will be parameterized by variable $\Lambda = \frac{1}{2\pi}\omega t$, called reduced time [4] or reduced crystal thickness [5]. Here variable

$$\omega = \sqrt{\frac{\partial_x^2 V(x)}{m_\mathrm{p}}}\Bigg|_{x=0}, \tag{11}$$

represents an angular frequency of proton trajectories in the center of the potential well.

For fixed value of Λ trajectory family define maps of the particle starting position b to its current position denoted $X(b) \equiv x(\Lambda; b)$, and to its current scattering angle denoted $\Theta_x(b) \equiv \theta_x(\Lambda; b)$. Their critical sets consist of all points satisfying equations $\partial_b X(b) = 0$, and $\partial_b \Theta_x(b) = 0$, which are called spatial and angular rainbow points, respectively. Note that when rainbow points exist inverse maps $X \to b$ and $\Theta_x \to b$ are multivalued, therefore singular. Considered together maps $X(b)$ and $\Theta_x(b)$ define a curve in the phase space $\mathcal{R} = (Q(b), P(b))$, called a rainbow diagram, whose critical points are spatial and angular rainbows [6]. In the case of regular dynamics, the rainbow diagram is also known as a whorl [7].

Trajectories of critical points $\partial_b x(\Lambda; b) = 0$, and $\partial_b \theta_x(\Lambda; b) = 0$ form lines called caustics [8]. They are identical to envelope lines associated with the trajectory family [9]. The density of trajectories is infinite at caustic lines, therefore a probability for finding a classical particle on caustic lines is very large. The caustic pattern is structurally stable [10–12]. This means that it can be modeled locally by the appropriate catastrophic prototype of codimension one

$$A_k(\eta; c_1, c_2, \ldots, c_{k-1}) = \frac{1}{k+1}\eta^{k+1} + \frac{c_{k-1}}{k-1}\eta^{k-1} + \cdots + \frac{c_2}{2}\eta^2 + c_1\eta. \tag{12}$$

In quantum mechanics, it is meaningless to speak about actual values of position and scattering angle particle might have. Instead, a state of the system is specified by a wave function $\Psi(\boldsymbol{r}, t)$ which in its own right determines only probability distributions of physical quantities. In analogy with the classical case, the energy loss of channeled particles will

be neglected. Evolution of the quantum state is then given by the following Schrödinger equation

$$i\hbar\partial_t\Psi(\boldsymbol{r},t) = \left[-\frac{\hbar^2}{2m_{\rm p}}\nabla^2 + V(x)\right]\Psi(\boldsymbol{r},t), \tag{13}$$

with initial condition of the form

$$\Psi_0(\boldsymbol{r}) = \psi_0(x)\zeta_0(y)\exp[ik_z z], \tag{14}$$

here k_z represents proton's initial wave vector ($E_k = \hbar k_z^2/2m_{\rm p}$), while $\psi_0(x)\zeta_0(y)$ is the transverse part of the initial wave function. The dependence of the potential (6) only on the coordinate x makes motions in the y, and z directions free. Therefore, a general wave function can always be represented in the form

$$\Psi(\boldsymbol{r},t) = \psi(x,t)\int_{\bar{y}} K(y,\bar{y},t)\zeta_0(y)\mathrm{d}\bar{y}\exp[ik_z z - iE_k t/\hbar], \tag{15}$$

and $K(y,\bar{y})$ is the free space propagator

$$K(y,\bar{y},t) = \sqrt{\frac{m_{\rm p}}{2\pi i\hbar t}}\exp\left[-\frac{m_{\rm p}}{2i\hbar t}(y-\bar{y})^2\right]. \tag{16}$$

As a result, an evolution of the transverse wave function $\psi(x,t)$ is governed by the reduced Schrödinger equation

$$i\hbar\partial_t\psi(x,t) = \left[-\frac{\hbar^2}{2m_{\rm p}}\partial_x^2 + V(x)\right]\psi(x,t). \tag{17}$$

Since motion in y and z direction is trivial it will be disregarded from the subsequent analysis. The evolution of the quantum dynamics will be also parameterized by the variable Λ.

In quantum mechanics, the closest analog of a classical particle is a wave packet. We have assumed that initial wave function is a Gaussian function

$$\psi_0(x) = \frac{1}{\sqrt{\sqrt{2\pi\sigma_x^2}}}\exp\left[-\frac{(x-b_0)^2}{4\sigma_x^2}\right], \tag{18}$$

of mean value b_0, and standard deviation σ_x. The wave function in the angular representation $\varphi(\theta_x,\Lambda)$ is defined by an integral

$$\varphi(\theta_x,\Lambda) = \sqrt{\frac{k_z}{2\pi}}\int\psi(x,\Lambda)\exp\left[-ik_z\theta_x x\right]\mathrm{d}x. \tag{19}$$

The initial wave function in the angular representation is therefore also Gaussian function

$$\varphi_0(x) = \frac{1}{\sqrt{\sqrt{2\pi\sigma_\theta^2}}}\exp\left[-\frac{\theta_x^2}{4\sigma_\theta^2} - ik_z\theta_x b_0\right]. \tag{20}$$

Standard deviations σ_x and σ_θ are linked through Heisenberg's uncertainty relation $k_z\sigma_\theta\sigma_x = 1/2$.

Hamilton's principal functions in the spatial and angular representations are defined by equations

$$\frac{\mathrm{d}}{\mathrm{d}x}S_x(x) = \hbar k_z \theta_x(x), \quad \text{and} \quad \frac{\mathrm{d}}{\mathrm{d}\theta x}S_\theta(\theta_x) = -\hbar k_z x(\theta_x). \tag{21}$$

Let us introduce reduced principal functions $\bar{S}_x$ and $\bar{S}_\theta$ by relations $S_x(x) = \hbar k_z \bar{S}_x(x)$, and $S_\theta(\theta_x) = -\hbar k_z \bar{S}_\theta(\theta_x)$, respectively. Semiclassical wave functions in the initial value representation [13, 14] are then given by integrals

$$\begin{aligned} \psi(x,\Lambda) &= \frac{1}{\sqrt{2\pi}}\int |\psi_0(b)| \exp\left[-ik_z\left(\bar{S}_\theta(b) + x\Theta_x(b)\right)\right]\sqrt{\frac{\mathrm{d}\Theta_x(b)}{\mathrm{d}b}}\mathrm{d}b, \\ \varphi(\theta_x,\Lambda) &= \frac{1}{\sqrt{2\pi}}\int |\psi_0(b)| \exp\left[ik_z\left(\bar{S}_x(b) - \theta_x X(b)\right)\right]\sqrt{\frac{\mathrm{d}X(b)}{\mathrm{d}b}}\mathrm{d}b, \end{aligned} \tag{22}$$

where $\mathrm{d}\bar{S}_x(b) = \Theta_x(b)\mathrm{d}X(b)$ and $\mathrm{d}\bar{S}_\theta(b) = X(b)\mathrm{d}\Theta_x(b)$. Note that phase functions are defined on the interval of the length d_{111}. So that integrands can be considered as rapidly oscillating functions, and semiclassical representation (22) valid, it is necessary that $k_z d_{111} \gg 1$.

The proton beam is represented as an ensemble of noninteracting wave packets where states $\psi_b(x,\Lambda)$ forming the ensemble are parameterized by the impact parameter b. According to the rules of the quantum mechanics state of the ensemble is specified by the density matrix operator $\hat{\rho}$, whose spatial and angular representations, ρ_x, and ρ_θ, are given by relations

$$\rho_x(x,\Lambda) = \sum_b p_b|\psi_b(x,\Lambda)|^2, \quad \rho_\theta(\theta_x,\Lambda) = \sum_b p_b|\varphi_b(\theta_x,\Lambda)|^2, \tag{23}$$

respectively. Expansion coefficient p_b represents relative frequency of the state ψ_b in the ensemble ($\sum_b p_b = 1$). Note that the assumption of an absolutely parallel beam is incompatible with the representation of particles as wave packets. Therefore, we assume that the incoming beam has a Gaussian profile of very small angular divergence Ω. Unknown parameters σ_θ σ_x, the distribution of the impact parameters, and their statistical weights p_b, should be determined in such way that $\rho_x(x,0)$ is uniform distribution in the interval of length d_{111}, while $\rho_\theta(\theta_x,0)$ is

$$\rho_\theta(\theta_x,0) = \frac{1}{\sqrt{2\pi\Omega^2}}\exp\left[-\frac{\theta_x^2}{2\Omega^2}\right]. \tag{24}$$

Using Eqs. (20) and (23) it is easy to show that $\sigma_\theta = \Omega$, and $\sigma_x = 1/(2k_z\Omega)$. An 1D grid of M impact parameters was taken to uniformly cover the interval $-d_{111}/2 \leq x \leq d_{111}/2$, with $p_b = 1/M$. The number M is minimal for which the difference between $\rho_x(x, 0)$ and the uniform distribution is smaller than some predetermined quantity.

In the (x, θ_x) phase space Wigner function associated with the state ψ_b is given by the following expression [15]

$$W_b(x, \theta_x) = \frac{k_z}{2\pi} \int \psi_b^\dagger \left(x - \frac{\xi}{2} \right) \psi_b \left(x + \frac{\xi}{2} \right) \exp\left[-ik_z\theta_x\xi \right] \mathrm{d}\xi, \tag{25}$$

or equivalently by

$$W_b(x, \theta_x) = \frac{k_z}{2\pi} \int \varphi_b^\dagger \left(\theta_x + \frac{\xi}{2} \right) \varphi_b \left(x - \frac{\xi}{2} \right) \exp\left[ik_z x\xi \right] \mathrm{d}\xi, \tag{26}$$

here † stands for Hermitian conjugation. A Wigner function of the ensemble is given by the expression

$$W(x, \theta_x) = \sum_b p_b W_b(x, \theta_x). \tag{27}$$

It is the closest quantum analog of the classical probability density in the phase space. The Wigner function has many properties out of which the following

$$\begin{aligned} &\int W(x, \theta_x)\mathrm{d}x = \rho_\theta(\theta_x), && \int W(x, \theta_x)\mathrm{d}\theta_x = \rho_x(x), \\ &W^\dagger(x, \theta_x) = W(x, \theta_x), && -\frac{k_z}{\pi} \leq W(x, \theta_x) \leq \frac{k_z}{\pi}, \end{aligned} \tag{28}$$

are important for the subsequent analysis. It should be stressed that in the classical limit Wigner function is given by the relation [16, 17]

$$W(x, \theta_x) = \frac{\delta(x - X(b), \theta_x - \Theta_x(b))}{\int_b \sqrt{(\partial_b X(b))^2 + (\partial_b \Theta_x(b))^2 \mathrm{d}b}}, \tag{29}$$

which value is 0 except for the points belonging to the rainbow diagram $\mathcal{R} = (Q(b), P(b))$.

Multiplicities of the maps $X \to b$ and $\Theta_x \to b$ are responsible for the enhancement of the wave packet self-interference in the vicinity of the classical caustic lines, and rainbow diagram in the phase space [4, 11, 18]. Structurally stable structures also appear in the quantum domain. They are locally isomorphic to the canonical diffraction patterns defined by the relation [11, 12]

$$\chi_k(c_1, c_2, \ldots, c_{k-1}) = \frac{1}{\sqrt{2\pi}} \int \exp\left[iA_k(\eta; c_1, c_2, \ldots, c_{k-1}) \right] \mathrm{d}\eta. \tag{30}$$

B. The proton energy loss

We are investigating the dynamics of the protons channeling in the [111] channel of the tungsten crystal in the interval $0 \leq \Lambda \leq 2$. Lattice constant of the tungsten crystal is $a_L = 0.3165$ nm, and number of atoms per unit cell is $N_a = 8$, giving interplanar distance $d_{111} = 0.0914$ nm, and planar surface density $\sigma_{111} = 5.7636$ nm^{-2}. Screening length was found to be $a_{\mathrm{s}} = 11.2$ pm. At the room temperature $T = 301$ K, amplitude of the thermal vibrations is $\sigma_{\mathrm{th}} = 5$ pm. It was found that $N = 3$ is sufficient for convergence of the continuous potential (6). For 2 MeV protons, the critical channeling angle is $\Theta_c = 5.9618$ mrad, while $\Lambda = 2$ corresponds to the crystal thickness of $L = 107.4027$ nm.

According to the Ziegler-Biersack-Littmark theory, the average energy loss of 2-MeV protons in tungsten crystal is dominated by electronic energy loss. Traversing crystal in the random direction protons will lose on average $\Delta E_k = 10.101$ keV. Average energy loss of the planary channeled protons differ by the factor $\nu \approx 0.35$ called the relative energy loss [19, 20], giving average energy loss of $\delta E_k = \nu \Delta E_k = 3.5354$ keV, which is utterly negligible. For considered crystal thickness, the average variance of the proton scattering angle fluctuations given by the relation

$$\delta\Omega_c^2 \approx \frac{m_{\mathrm{e}}}{4m_{\mathrm{p}}} \frac{\delta E_k}{E_k} = 0.4906 \text{ mrad}, \tag{31}$$

is sufficiently small that the dechanneling process can be neglected.

Our decision to neglect both effects can be justified on the following grounds. First, the electron density is not uniform in the region of the channel. Its maximal value at the atomic plane is approximately 10 times larger than its minimal value at the center of the channel. Energy loss and dispersion of the scattering angle of the well-channeled protons are grossly overestimated since particles spend only a fraction of time in regions of high electron density. Fluctuations of scattering angle decrease the sharpness of the observed structures and limit the resolution of measured distributions (see section C). This is a serious limitation of any distribution recording system based on image detection. However, modern particle counting detectors based on the microchannel plate technology offer a very large dynamical range. In the pulsed operation, they can provide reliable count rates up to 1GHz [21], meaning that dynamical range is limited only by available time to accumulate sufficient statistics.

Second, in this study, we are primarily investigating to which extent coordinated self-interference of wave packets is responsible for the formation of patterns on a global scale.

It will be shown when coordinated self-interference amplifies the classical aspects and when it amplifies wave-like aspects of the ensemble dynamics. The mentioned processes are unaffected by the slight inaccuracies of the obtained results on the smallest scale.

C. Influence of the angular divergence on the probability densities

Let us consider the influence of the fluctuation of the scattering angle on the angular distribution $Y(\theta)$. Fluctuations may be caused by the angular divergence of the incoming beam, interaction with the crystal electrons, thermal vibrations *etc.*. It is convenient to Fourier analyse the function $Y(\theta)$ in the harmonic components $\exp\left(i\frac{2\pi}{\vartheta}\theta\right)$ and analyse influence of the fluctuation on each component separately. If distribution of the fluctuations is Gaussian of variance Ω_c^2, then modified spectral component $I_\vartheta(\theta)$ is given by the integral

$$I_\vartheta(\theta) = \int \frac{1}{\sqrt{2\pi\Omega_c^2}} \exp\left[-\frac{(\theta-\theta')^2}{2\Omega_c^2}\right] \exp\left(i\frac{2\pi}{\vartheta}\theta'\right) \mathrm{d}\theta', \tag{32}$$

which can be evaluated analytically giving

$$I_\vartheta(\theta) = \exp\left[-\frac{2\pi^2\Omega_c^2}{\vartheta^2}\right] \exp\left(i\frac{2\pi}{\vartheta}\theta\right). \tag{33}$$

Therefore, fluctuations of scattering angle produce exponential damping of all spectral components which is especially severe for $\vartheta < \Omega_c$. As a result, the *sharpness* of structures in the resulting distribution $Y(\theta)$ is depreciated. This does not mean that damping erases all structures smaller than Ω_c. What structures will be visible depends on the dynamical range of the used detector. For example to see structures of the same size as Ω_c an amplification of the signal need to be at least $\exp(2\pi^2)$ or 85.7263 dB, which is in the range of the modern electronic. On the other hand, detection of the structures of the size $\Omega_c/2$ requires a dynamical range of 342.9052 dB.

D. Catastrophic modeling of caustics

We wish to model a caustic line from the first rainbow cycle by a bifurcation set of the A_5 catastrophe which is defined by the relation

$$A_5 = \frac{1}{6}\eta^6 + \frac{c_4}{4}\eta^4 + \frac{c_3}{3}\eta^3 + \frac{c_2}{2}\eta^2 + c_1\eta. \tag{34}$$

Here, state variable η is taken to be proportional to the impact parameter b, while values of parameters $c_1, \ldots, c_4$ are to be determined. The critical points and degenerate critical points of polynomial A_5 are solutions of equations

$$\begin{aligned} \frac{\mathrm{d}A_5}{\mathrm{d}\eta} &= \eta^5 + c_4\eta^3 + c_3\eta^2 + c_2\eta + c_1 = 0, \\ \frac{\mathrm{d}^2A_5}{\mathrm{d}\eta^2} &= 5\eta^4 + 3c_4\eta^2 + 2c_3\eta + c_2 = 0. \end{aligned} \tag{35}$$

The first of Eqs. (35) defines the function

$$c_1 = -\eta^5 - c_4\eta^3 - c_3\eta^2 - c_2\eta, \tag{36}$$

which we assume to be proportional to the spatial deflection function $X(b)$. Because of symmetry $c_3 = 0$. In that case the second of Eq. (35) can be solved giving

$$\eta^2 = -\frac{3c_4}{10}\left(1 \mp \sqrt{1 - \frac{20}{9}\frac{c_2}{c_4^2}}\right). \tag{37}$$

Note that only for $c_4 < 0$ catastrophe A_5 has the correct multiplicity of real critical points. For $-\infty < c_2 < 0$ there are only two real critical points. When $c_2 = 0$ there is one doubly degenerate critical point $\eta = 0$, and critical point pair $\eta = \pm\sqrt{-c_4}$. For $0 < c_2 < 9c_4^2/20$ there are four critical points. When $c_2 = 9c_4^2/20$ there are two double degenerate points $\eta = \sqrt{-3c_4/10}$ and $\eta = -\sqrt{-3c_4/10}$, while for $c_2 > 9c_4^2/20$ there are no real critical points.

Variable c_2 behaves as an unfolding parameter, and we shall take it to be proportional to the variable Λ. The degenerate cryptical points from three cusps in the (c_1, c_2) space. Coordinates of their apexes are: $(\pm 3/25\sqrt{6c_4^5/5}, 9c_4^2/20)$, and $(0,0)$. In the (Λ, x) space coordinates of respective cusps of the caustic line are $(0.1598, \pm 0.01126\text{nm})$, and $(0.25, 0\text{nm})$ which occur for impact parameters $b = \pm 0.0326$ nm, and $b = 0$ nm. Taking into the account introduced relations $c_1 = x/x_0$, $c_2 = (\Lambda - \lambda_0)/\Lambda_0$, and $\eta = b/b_0$, unknown values of parameters were found to be $c_4 = -0.3742$, $x_0 = 1$ nm, $b_0 = 0.0988$ nm, while $\Lambda_0 = -1.4315$ and $\lambda_0 = 0.25$.

Bifurcation set of the obtained model is shown in Fig. 1(a). For $c_4 < 0$ unfolding of the catastrophe A_5 proceeds as follows. For $\Lambda < 0.16$ there are no degenerate critical points. For $\Lambda = 0.16$ two double degenerate critical points appear forming apexes of the cusps. For $\Lambda > 0.16$ degeneracy is lifted and apex points split into two pairs of degenerate cryptical points. This behavior is typical of the cusp catastrophe A_3, while the evolution

of the single degenerate cryptical points is equivalent to the behavior of the catastrophe A_2. Therefore, it could be said that catastrophe A_3 brakes into two A_2 catastrophes. Outer branches emanating out of cusp points move towards the channel axis where they meet for $\Lambda = 0.25$. Two A_2 catastrophes merge to form a new A_3 catastrophe and then disappear. Inner branches cross the channel axis and become outer branches. They are only degenerate critical points that exist for $\Lambda > 0.25$. The described unfolding is schematically represented in Fig. 1(b)

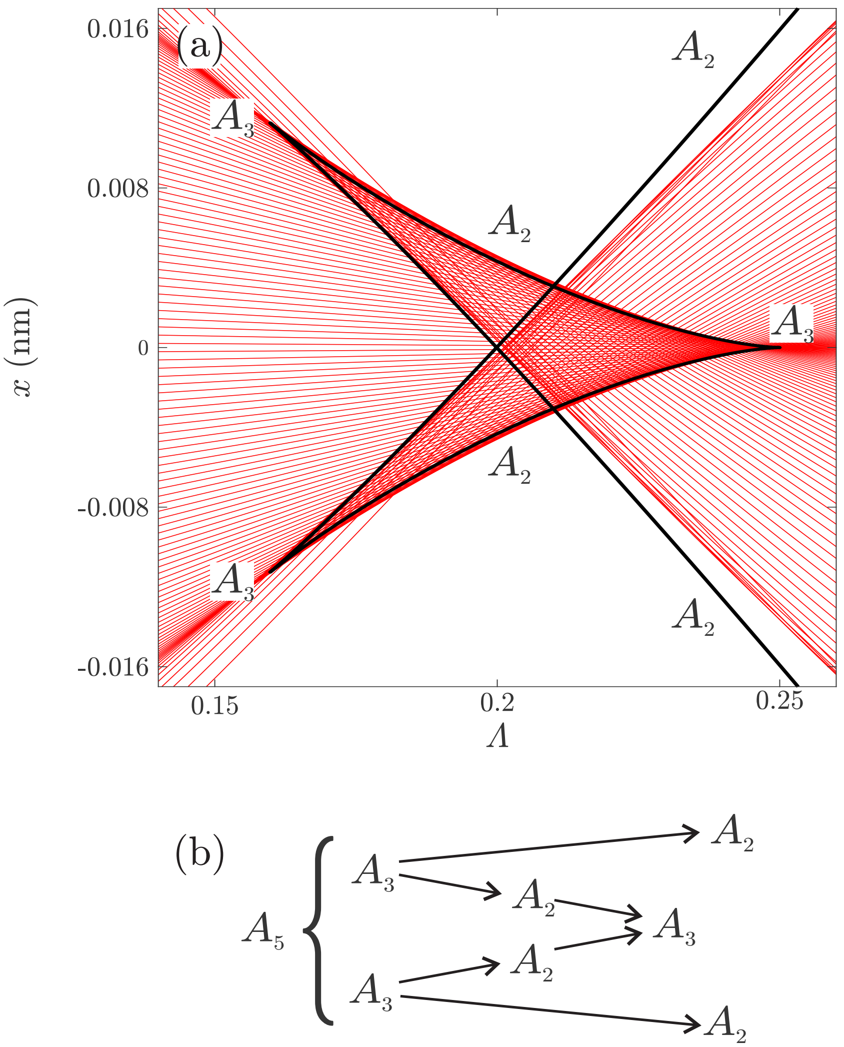

FIG. 1. (a) Enlarged view of the trajectory family encompassing the first rainbow cycle. The black line shows a model of the caustics by the bifurcation set of the A_5 catastrophe. (b) A diagram showing the unfolding of the catastrophe A_5 for variation of parameter Λ.

E. Semiclassical Wigner functions

For $\Lambda = 2$ spatial and angular deflection functions are in the vicinity of the impact parameter $b_0 = d_{111}/4$ approximated by the following polynomials

$$\begin{aligned} \Theta_x(b) &= p_1 b + p_0, \\ X(b) &= \frac{q_2}{2} b^2 + q_1 b + q_0, \end{aligned} \tag{38}$$

where $p_0 = 2.8294$ mrad, $p_1 = -233.9036$ mrad/nm, $q_0 = -0.0592$ nm, $q_1 = 7.8651$, $q_2 = -210.2381$ nm^{-1}. The corresponding reduced angular Hamilton's principal function is then given by the following polynomial

$$\bar{S}_\theta(b) = \frac{r_3}{3} b^3 + \frac{r_2}{2} b^2 + r_1 b + r_0. \tag{39}$$

To calculate the Wigner function of the Gauss-like ensemble corresponding to the impact parameter b_0 it is much more convenient to use integral representation Eq. (26) together with Eq. (22). Because of a rapid decrease of the initial distribution (14) validity of Eqs. (38) and (43) can be extended to infinity. By appropriate substitutions, it is possible to transform obtained integrals to the form [22]

$$\int_{-\infty}^{\infty} \left[i \left(\frac{1}{3} u^3 + a u^2 + b u \right) \right] = 2\pi \exp\left[i a \left(\frac{2}{3} a^2 - b \right) \right] \mathrm{Ai}(b - a^2). \tag{40}$$

Thus, the resulting Wigner function becomes

$$\begin{aligned} W_b(x, \theta_x) = {} & \frac{\gamma_1}{\sqrt{2\pi\sigma_x^2}} \exp\left[\frac{1}{\gamma_2} \left(\frac{1}{12\gamma_2 k_z^2 \sigma_x^2} - (p_1 x + r_1) - \frac{r_2}{p_1}(\theta_x - p_0) \right) \right] \\ & \times \chi_2 \left[\gamma_1 \left(\frac{1}{8\gamma_2 k_z^2 \sigma_x^2} - (p_1 x + r_1) + \frac{r_2}{p_1}(p_0 - \theta_x) + \frac{r_3}{p_1^2}(p_0 - \theta_x)^2 \right) \right], \end{aligned} \tag{41}$$

where $\gamma_1 = \sqrt[3]{4k_z^2/r_3}$, and $\gamma_2 = 2 r_3 \sigma_x^2$ are dimensionless parameters, while χ_2 is Airy's function of the first kind.

In the case of the plane-wave-like ensemble, the initial distribution is approximated with the uniform distribution. For $\Lambda = 0.6$ angular and spatial deflection functions can be approximated by the following odd polynomials

$$\begin{aligned} \Theta_x(b) &= p_1 b, \\ X(b) &= \frac{q_5}{5} b^5 + \frac{q_3}{3} b^3 + q_1 b, \end{aligned} \tag{42}$$

with parameters $p_1 = 121.87$ mrad/nm, $q_1 = -1.19$ nm, $q_3 = 1630$ nm^{-2}, and $q_5 = -571000$ nm^{-4}. The reduced angular Hamilton's principal function is given by polynomial

$$\bar{S}_\theta(b) = \frac{r_6}{6} b^6 + \frac{r_4}{4} b^4 + \frac{r_2}{2} b^2 + r_0. \tag{43}$$

Since the dominant contribution to the integral (26) comes from stationary points of the phase function in the region of one unit cell validity of the polynomial approximations (42) was extended to the infinity giving

$$W_b(x,\theta_x) = -\frac{\zeta}{2\pi d_{111}}\chi_4\left[\zeta\left(p_1 x + r_2\frac{\theta_x}{p_1} + r_4\frac{\theta_x^3}{p_1^3} + r_6\frac{\theta_x^5}{p_1^5}\right), 0, \zeta^3\left(\frac{3}{4}r_4\frac{\theta_x}{p_1} + \frac{5}{2}r_6\frac{\theta_x^3}{p_1^3}\right)\right], \quad (44)$$

where $\zeta = \sqrt[5]{16k_z^4 p_1/5s_6\theta_x}$ is an auxiliary function, while χ_4 is the swallowtail canonical diffraction pattern.

---

[1] G. Molière, Z. Naturforsch. **2a**, 133 (1947).

[2] D. S. Gemmell, Rev. Mod. Phys. **46**, 129 (1974).

[3] M. Abramowitz and I. Stegun, *Handbook of mathematical functions* (National Bureau of Standards, 1972) page 302.

[4] M. Ćosić, S. Petrović, and S. Bellucci, Chaos (2020), (submitted for publication).

[5] H. F. Krause, J. H. Barrett, S. Datz, P. F. Dittner, N. L. Jones, J. Gomez del Campo, and C. R. Vane, Phys. Rev. A **49**, 283 (1994).

[6] S. Petrović, M. Ćosić, and N. Nešković, Phys. Rev. A **88**, 012902 (2013).

[7] M. V. Berry, N. L. Balazs, M. Tabor, and A. Voros, Ann. Phys. **122**, 26– (1979).

[8] M. V. Berry and C. Upstill, *Catastrophe optics: morphologies of caustics and their diffraction patterns*, E. Wolff, Progress in Optics XVIII (North Holland, Amsterdam, 1980) pp. 257—346.

[9] J. W. Bruce and P. J. Giblin, *Curves and singularities* (Cambridge University Press, Cambridge, 1984).

[10] M. Ćosić, N. Nešković, and S. Petrović, Nucl. Instrum. Methods Phys. Res. Sect. B **444**, 10 (2019).

[11] M. V. Berry and K. V. Mount, Rep. Prog. Phys. **35**, 315 (1972).

[12] T. Poston and I. Stewart, *Catastrophe: Theory and Its Applications* (Pitman Publishing, London, 1978).

[13] W. H. Miller, J. Chem. Phys **53**, 1949 (1970).

[14] K. G. Kay, Annu. Rev. Phys. Chem. **56**, 255 (2005).

[15] E. Wigner, Phys. Rev. **40**, 749 (1932).

[16] M. V. Berry, Phil. Trans. R. Soc. A **287**, 237 (1977).

[17] G. A. Baker, Phys. Rev. **109**, 2198 (1958).

[18] M. V. Berry and N. L. M. Balazs, J. Phys. A: Math. Gen. **12**, 624 (1979).

[19] B. R. Appleton, C. Erginsoy, and W. M. Gibson, Phys. Rev. **161**, 330 (1967).

[20] M. B. H. Breese, P. J. C. King, G. W. Grime, P. J. M. Smulders, L. E. Seiberling, and M. A. Boshart, Sov. Phys. JETP **46**, 871 (1977).

[21] D. J. Gershman, L. A. Avanov, D. J. Chornay, A. C. Rager, C. J. Pollock, G. Grubbs, D. A. Mackler, C. J. Tucker, and N. P. Paschalidis, Rev.Sci.Instrum **98**, 073301 (2018).

[22] O. Valle'e and M. Soares, *Airy Functions and Applications to Physics* (World Scientific Publishing, 2004) page 10.
